# Identification of NIBAN2‐Regulated *RUNX2* Alternative Splicing Presents Novel Strategies for Antagonizing Osteoporosis

**DOI:** 10.1002/advs.202416536

**Published:** 2025-03-07

**Authors:** Sheng Zhang, Zhiqiang Yang, Yuanlong Xie, Yufeng Zhang, Zhe Chen, Xuan Lv, Zhouming Deng, Zan Huang, Lin Cai, Renxiong Wei

**Affiliations:** ^1^ Department of Spine Surgery and Musculoskeletal Tumor Zhongnan Hospital of Wuhan University No. 163 Donghu Road Wuhan Hubei 430071 P. R. China; ^2^ College of Life Sciences Key Laboratory of Cell Hemostasis of Hubei Province Wuhan University No. 299 Bayi Road Wuhan Hubei 430072 P. R. China; ^3^ Department of Orthopedics The Second Hospital of Tianjin Medical University No.23 Pingjiang Road Tianjin 300211 China

**Keywords:** alternative splicing, bone mineralization, osteoblast differentiation, osteoporosis, RUNX2

## Abstract

Osteoporosis is characterized by excessive bone resorption and/or defects in bone formation. Identification of factors promoting osteoblast differentiation may provide potential targets for osteoporosis therapy. Through integral analyses of multiple datasets, NIBAN2 is found to be tightly associated with bone formation and osteoporosis. Indeed, NIBAN2 promotes osteoblast differentiation, and conditional Niban2 knockout in osteoblasts caused bone loss and insufficient mineralization. Mechanistically, NIBAN2 interacts with the HNRNPU‐cored spliceosome complex and alters its components to regulate the alternative splicing of *RUNX2*, which ultimately cause an increase in functional *RUNX2* (nuclear localization sequence complete) but a decrease in dysfunctional *RUNX2* (exon 6 exclusive) to reinforce osteoblast differentiation. Most importantly, NIBAN2 expression level negatively correlates with *RUNX2* spliced isoforms and bone loss in osteoporosis patients. NIBAN2 overexpression rescues bone loss in ovariectomized mice. Thus, this research identifies NIBAN2‐regulated *RUNX2* alternative splicing as a potential mechanism of osteoblast differentiation that may present strategies for antagonizing osteoporosis.

## Graphical abstract

NIBAN2 interacts with the HNRNPU‐cored spliceosome complex and alters its components to regulate the alternative splicing of RUNX2, which ultimately cause an increase in functional RUNX2 (nuclear localization sequence complete) but a decrease in dysfunctional Runx2 (exon 6‐exclusive) isoforms to reinforce osteoblast differentiation. This presents potential strategies for antagonizing osteoporosis.

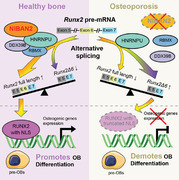

## 1 Introduction

Osteoporosis is characterized by skeletal fragility and microarchitectural deterioration with an increased risk of fragility fractures, which is the most common metabolic diseases of the skeletal system, causing a major economic burden worldwide.^[^ ^1^, ^2^, ^3^ ^]^ Most primary osteoporosis is attributed to excessive bone resorption and/or defects in bone formation.^[^ ^4^ ^]^ In recent years, the application of anabolic agents, including teriparatide (ligand binding to parathyroid hormone receptor type 1) and romosozumab (humanized monoclonal antibody to sclerostin), has improved osteoporosis treatment. However, such treatment is limited due to its short duration as well as rapid bone loss and an increased risk of fractures due to discontinuation,^[^ ^5^, ^6^ ^]^ which urges for new anabolic targets.

Osteoblasts (OBs) are mainly derived from skeletal stem cells (previously described as mesenchymal stem cells) via OB differentiation,^[^ ^7^, ^8^ ^]^ which determines the rate of bone formation.^[^ ^9^ ^]^ Osteoblast differentiation involves complicated regulation of cytokine signaling, transcription factors, and epitranscriptomic modifications.^[^ ^10^, ^11^ ^]^ For instance, runt‐related transcription factor 2 (RUNX2) is the master regulator of osteogenesis.^[^ ^8^ ^]^ Multiple signaling pathways, including WNT and FGF, induce RUNX2 expression or activate RUNX2, which further stimulates the expression of downstream osteogenic genes to reinforce OB differentiation.^[^ ^12^ ^]^ Notably, RUNX2 function in OB differentiation is regulated by alternative splicing (AS).^[^ ^13^, ^14^, ^15^ ^]^ The expression of a specific isoform contributes to cell type specification by activating isoform‐specific transcriptional targets or antagonizing the function of the full‐length protein.^[^ ^14^ ^]^ Furthermore, some AS transcripts skipping the critical exons encode isoforms with incorrect subcellular localization or without functional domains that result in null function or repression of OB differentiation.^[^ ^14^ ^]^ However, the further mechanism of *RUNX2* AS and whether it could be targeted for osteoporosis therapy have not been addressed.

In this research, we discovered that NIBAN2 (niban apoptosis regulator 2, also known as MINERVA or FAM129B) is a novel factor that promotes OB differentiation. NIBAN2 is a member of the FAM129 protein family, which is involved in key signaling pathways regulating cell survival, proliferation, and apoptosis. NIBAN2 is upregulated in many types of cancers and promotes invasion.^[^ ^16^, ^17^, ^18^ ^]^ However, the function of NIBAN2 in OB differentiation and osteoporosis is not known. We found that NIBAN2 was tightly associated with OB differentiation and osteoporosis. NIBAN2 promoted OB differentiation and rescued bone loss in an osteoporosis mouse model. Furthermore, we unveiled a novel mechanism by which NIBAN2 regulated AS of *RUNX2* by binding to HNRNPU and switching the components of the HNRNPU‐cored spliceosome complex. Above posttranscriptional gene regulation ultimately caused an increase in functional RUNX2 (nuclear localization sequence complete) but a decrease in dysfunctional RUNX2 (exon 6‐exclusive) isoforms to reinforce osteoblast differentiation. Our research identifies NIBAN2 as a potent factor promoting OB differentiation by altering *RUNX2* splicing. This work also provides a potential anabolic target for osteoporosis therapy.

## 2 Results

### 2.1 Identification of NIBAN2 as a Marker Gene in the Osteoblast Cluster with High Osteogenic Activity in scRNA‐Seq

**Figure 1:**
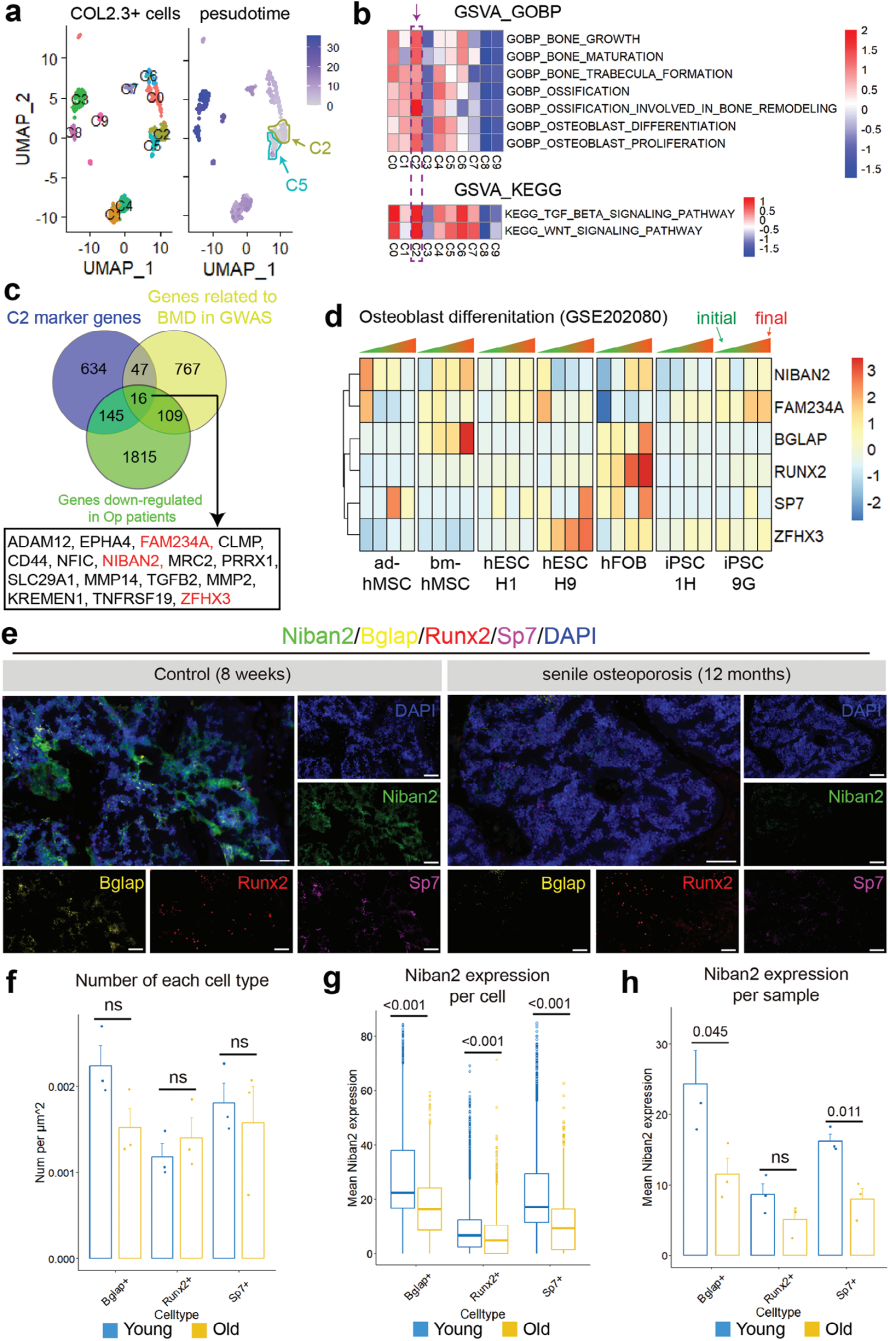
Identification of a high osteogenic activity osteoblast cluster in scRNA‐seq with the clinical‐related marker gene *NIBAN2*. a) UMAP reduction blot and pseudotime trajectory inference analysis of the osteoblast scRNA‐seq dataset. b) GSVA of the bone formation pathway enrichment levels in each OB cluster. c) Venn blot of OB cluster 2 marker genes with BMD‐related genes in GWAS and downregulated genes in osteoporosis patients. Unreported genes in bone research are marked in red. d) Heatmap of *ZFHX3*, *FAM234A*, *NIBAN2*, and OB markers expression tendency during OBs differentiation in different cell type. e) Multiplex immunofluorescence of Niban2 expression in different celltypes of mice with senile osteoporosis and controls (scale bar: 50 µm). Representative images for 3 independent samples. f) Quantitative analysis of cell number in panel e. Quantitative analysis of mIF in panel b with Niban2 expression levels in the g) single‐cell level or h) mean sample level. Data are presented as the mean (SD), except the boxplot, and individual data are indicated as points. Data in boxplot are presented as quarters with points of outliers. *P* values were tested by unpaired Student's *t‐*test. *p* values were presented if it < 0.05.

To identify essential factors regulating OB differentiation, we performed a comprehensive analysis integrating multiple datasets. Single‐cell transcriptome data of Col2.3^+^ cells (Type 1 collagen‐expressing cells) were extracted from GSM2915579, in which the non‐hematopoietic single‐cell bone marrow microenvironment landscape was constructed.^[^ ^1^ ^]^ To note, cells under stress in the original research were not included in the reanalysis. Based on transcriptional profiles, a total of 880 Col2.3^+^ cells were filtered and divided into 10 clusters (**Figure** **1**a), and different expression patterns of osteogenic marker genes were observed (Figure S1a, Supporting Information). Transcriptional states allowed us to generate a pseudotime trajectory, in which C2 and C5 cells were at the initial stages (Figure 1a). Notably, C1 and C4 cells were at the transitional stages with high levels of the mature OB markers *Bglap*, *Bglap2* (Figure S1a, Supporting Information). The osteogenic activity was further evaluated in each cluster by gene set variation analysis (GSVA) (Figure 1b). C2 OBs exhibited the highest enrichment both in bone formation‐related biological processes and OB differentiation‐related signaling pathways, including WNT and TGFβ signaling (Figure 1b). Moreover, 842 marker genes of cluster 2 were extracted for Venn diagram analysis with 939 bone mineral density (BMD)‐related genes identified by GWAS^[^ ^2^ ^]^ and 2085 downregulated genes in osteoporosis patients^[^ ^3^ ^]^ (GSE35958) (Figure 1c). In total, 16 genes were identified, of which 13 genes were known to be relevant to bone‐loss diseases or OB differentiation.^[^ ^4^ ^]^ Of the remaining 3 genes, *NIBAN2* was one of the marker genes in cluster 2 (Figure S1b, Supporting Information) and was up‐regulated during OB differentiation in this dataset (Figure S1 c, Supporting Information). Moreover, in another database (GSE202080), *NIBAN2* expression increased significantly during OB differentiation in bone marrow‐derived hMSCs and hFOB cells (a human pre‐osteoblasts cell line), rather than adipose‐derived hMSCs, hESCs, or iPSCs, which emphasized the correlation between *NIBAN2* and OB differentiation in bone‐derived lineage (Figure 1d). While the other two candidates, *FAM234A* and *ZFHX3*, exhibited less consistency with osteogenesis (Figure 1d). Thus, our integral analyses suggest that NIBAN2 may be a novel factor regulating OB differentiation.

### 2.2 Niban2 is Tightly Associated with Osteoporosis and Osteoblast Differentiation

To confirm the integrated bioinformatic analysis, we measured *Niban2* expression and confirmed its upregulation during OB differentiation in primary mouse cranial pre‐osteoblasts (pre‐OBs) (Figure S1 d, Supporting Information). Niban2 upregulation was verified during in vitro OB differentiation in pre‐OBs detected by western blots and immunofluorescence (Figures S1e–g, Supporting Information). Interestingly, Niban2 protein expression in the nucleus was gradually upregulated from 1 to 6 days (Figures S1d–f, Supporting Information). To further confirm the correlation between NIBAN2 and osteoporosis, we performed multiplex immunofluorescence (mIF) to explore its expression in different OB stages and senile osteoporosis mouse model (Figure 1e). Markers including Runx2, Sp7, and Bglap could roughly divide osteo‐lineage cells into early (Runx2+ or Sp7+) and late (Bglap+) stages. Although the number of each cell types exhibited no statistically calculated difference, the number of Bglap+ and Sp7+ cells in osteoporosis showed a decreasing tendency, which also implicated OB differentiation blockage in osteoporosis (Figure 1f). Niban2 displayed highest expression level in Bglap+ cells and decreased in all above cell types during senile osteoporosis in single cell level (Figure 1g) and biological replicates (Figure 1h). These results confirm our bioinformatic analysis and demonstrate a tight correlation of NIBAN2 with osteoporosis and OB differentiation in mice.

### 2.3 Niban2 Deficiency Causes Bone Loss and Insufficient Mineralization Due to Impaired Osteoblast Differentiation

**Figure 2:**
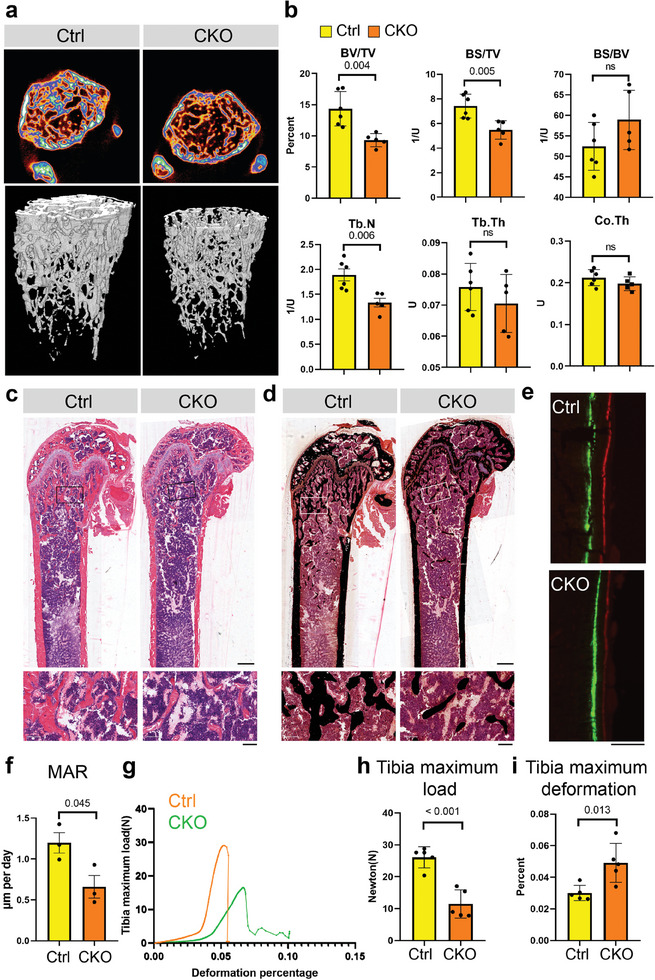
*Niban2* deficiency in OBs causes bone loss and insufficient mineralization in mice. a) Representative cross section and reconstruction images of *Niban2* CKO male mice and control mice in 12‐week by µCT. b) Quantitative analysis of the µCT results of panel a. c) Representative H&E staining of *Niban2* CKO mice and control mice (scale bar: 800 µm; for magnification, scale bar: 100 µm). d) Representative von Kossa staining of *Niban2* CKO mice and control mice (scale bar: 800 µm). e) Dual fluorochrome labeling of bone mineralization in *Niban2* CKO mice and control mice from 8 weeks to 12 weeks postnatally. Calcein (green label) was injected at 8 weeks, and Alizarin‐3‐methyliminodiacetic acid (red label) was injected at 12 weeks. Representative images for independent samples. f) Quantitative analysis of the fluorochrome label results in panel e. The mineral apposition rate (MAR) was calculated. g) Compressive mechanical test of *Niban2* CKO tibia and the controls. Representative images for independent samples. Quantitative analysis of the h) maximum load and i) maximum deformation in panel g. Data are presented as the mean (SD), and individual data are indicated as points. *p* values were tested by unpaired Student's *t*‐test. *p* values were presented if it < 0.05.

To investigate the in vivo function of NIBAN2 in bone, we generated OB‐lineage specific *Niban2* knockout mice by crossing *Niban2*‐floxed mice with *Bglap‐Cre* mice (*Bglap‐Cre*;*Niban2^flox/flox^*, hereafter named as CKO).^[^ ^5^ ^]^ The *Bglap‐Cre* was chosen based on our mIF since Bglap+ cells exhibited highest expression level of Niban2 and a decreasing tendency during osteoporosis (Figure 1e–h). Niban2 deficiency in OBs in femurs from CKO mice was confirmed by immunohistochemistry (Figure S2 a, Supporting Information). Morphologically, CKO male mice had no overt developmental defects in terms of body weight or body length compared to the control mice (Figure S2 b, Supporting Information). However, microcomputed tomography (µCT) imaging revealed that CKO male mice displayed fewer trabeculae in the distal femurs than the control mice at 12 weeks (**Figure** **2**a). In distal femoral trabecular bone, *Niban2* deficiency also significantly decreased bone volume per tissue volume (BV/TV), bone surface per tissue volume (BS/TV), and trabecular number (Tb. N) (Figure 2b). H&E staining further verified that CKO mice exhibited less trabecular bone than the control mice (Figure 2c). Moreover, von Kossa staining showed that CKO mice formed less mineral deposition in the distal femoral trabecular region and exhibited thinner cortical bone mineralization than the control mice (Figures 2d,e). Similar µCT and histology results were observed in 4‐week‐old CKO male mice (Figure S2c–g, Supporting Information) and 8‐week‐old CKO female mice (Figure S3 a,b, Supporting Information). Temporal fluorochrome labeling mineral deposition from 8 to 12 weeks demonstrated that *Niban2* deficiency significantly reduced the width of the fluorochrome‐labeled gap (Figure 2e), and the quantitative analysis confirmed a lower mineral apposition rate (MAR) in CKO mice (Figure 2f). Finally, the compressive mechanical test demonstrated that insufficient mineralization resulted in reduced mechanical peak load and stiffness in femurs from CKO mouse compared to those from control mice (Figure 2g–j, Supporting Information). These observations demonstrate that Niban2 deficiency in OB lineages causes bone loss due to insufficient mineralization and defect bone formation.

**Figure 3:**
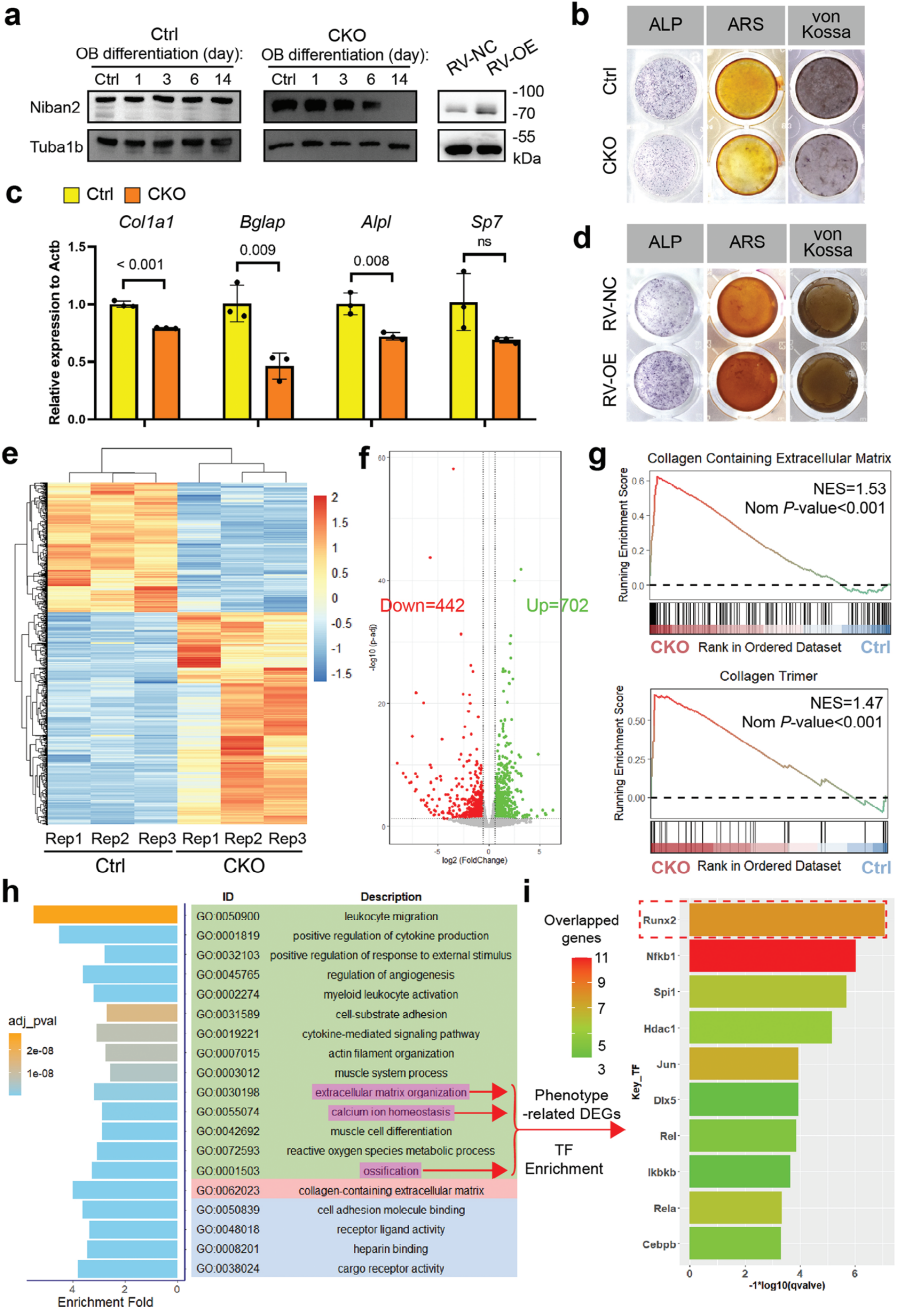
Niban2 promotes osteoblast differentiation by regulating Runx2. a) Western blot for Niban2 in *Niban2* CKO pre‐OBs and its control during OB differentiation. Western blot for Niban2 in *Niban2* overexpressing pre‐OBs and its control was also presented in this panel. b) OB differentiation staining of *Niban2* CKO pre‐OBs and the controls, including ALP, ARS, and von Kossa staining. c) The results of quantitative RT‒PCR to detect osteoblast differentiation marker gene expression, including *Col1a1*, *Alpl*, *Bglap*, and *Sp7*. d) *Niban2* overexpressing pre‐OBs and the controls. Including ALP, ARS, and von Kossa staining. e) Heatmap of differentially expressed genes (DEGs) in the transcriptome data of *Niban2* CKO pre‐OBs and the controls after 8 days of OB differentiation to induce Niban2 knockout. f) Volcano plot of the transcriptome data of *Niban2* CKO pre‐OBs and the controls. DEGs were marked green or red for downregulated and upregulated, respectively. g) GSEA result of extracellular matrix formation and collagen trimer in *Niban2* CKO pre‐OBs and control cells. h) GO bar plot illustrating GO enrichment results of DEGs in *Niban2* CKO pre‐OBs and the controls. i) TF enrichment analysis of phenotype‐related DEGs, the items of which are marked in panel h. Data are presented as the mean (SD), and individual data are indicated as points. *p* values were tested by unpaired Student's *t*‐test or one‐way ANOVA followed by Bonferroni's post hoc test. *p* values were presented if it < 0.05.

Impaired OB differentiation is one of the major factors causing defect bone formation. In support of these observations, two mineralization‐related proteins, Collagen I and Spp1,^[^ ^4d^ ^]^ were downregulated in the distal femoral trabecular regions of CKO mice compared to those of control mice (Figure S3c–e, Supporting Information), demonstrating OB differentiation defect in vivo. To further verify whether Niban2 deficiency caused defective OB differentiation leading to alterations in bone structure, we cultured pre‐OBs from CKO and control mice and induced OB differentiation. NIBAN2 protein expression was significantly downregulated in CKO cells 6 days after induction compared to its control (**Figure** **3**a). Alkaline phosphatase (ALP), alizarin red (ARS), and von Kossa staining showed that CKO cells exhibited ALP activity and fewer calcium deposits than the control cells with quantitative analysis (Figure 3b and Figure S3 f, Supporting Information). Consequently, multiple Runx2 target genes were downregulated in CKO pre‐OBs, including *Col1a1*, *Bglap*, *Alpl*, and *Sp7* (Figure 3c). Moreover, Niban2 overexpression (Figure 3a) promoted OB differentiation and calcium deposition, as evidenced by increases in ALP activity, ARS staining and von Kossa staining with quantitative analysis (Figure 3d and Figure S3 g, Supporting Information). These results suggest that NIBAN2 deficiency causes bone loss due to impaired OB differentiation.

Bone loss can also be caused by enhanced bone resorption due to osteoclast activation. Therefore, we evaluated whether osteoclasts might play a role in bone loss in CKO mice. Interestingly, we observed fewer TRAP‐positive osteoclasts (OC) in CKO mice than that in control mice (Figure S3 h, Supporting Information). In addition, in vitro osteoclast differentiation of bone marrow monocytes from CKO mice and control mice did not show significant differences (Figure S3 j,k, Supporting Information). These results exclude the possibility that the bone loss in CKO may be attributed to enhanced osteoclastogenesis.

NIBAN2 was previously shown to suppress apoptosis in many types of cells.^[^ ^6^ ^]^ However, Niban2 knockdown in pre‐OB cell lines did not significantly affect proliferation and apoptosis (Figure S4a–d, Supporting Information). Moreover, Niban2 deficiency in vivo also exhibited no significant difference on apoptosis assays, including TUNEL staining and detection of cleaved caspase‐3 (Figure S4e–h, Supporting Information).

Taken together, Niban2 deficiency causes bone loss and insufficient mineralization due to impaired OB differentiation. These observations unveil a novel role of Niban2 that positively regulates OB differentiation.

### 2.4 Niban2 Promotes Osteoblast Differentiation by Regulating Runx2 Alternative Splicing

To explore the mechanism underlying the impaired OB differentiation induced by *Niban2* deficiency, we performed RNA‐seq analysis on pre‐OBs derived from CKO and control mice. The heatmap illustrated the differential expression landscape with unsupervised clustering (Figure 3e). In total, 1144 differentially expressed genes (DEGs; fold change > 1.5, padj < 0.05) were identified, of which 442 genes were downregulated and 702 genes were upregulated in *Niban2* CKO pre‐OBs (Figure 3f). Gene Set Enrichment Analysis (GSEA) revealed alteration of extracellular matrix due to *Niban2* deficiency (Figure 3h). The bar plot showed that DEGs exhibited enrichment in multiple Gene Ontology (GO) terms, including “extracellular matrix organization.” “calcium ion homeostasis,” and “ossification” (Figure 3h). DEGs that were attributed to at least one of above three critical OB‐related processes were extracted and assigned as phenotype‐related DEGs. OB differentiation is driven by several key transcription factors (TFs).^[^ ^7^ ^]^ To ascertain which TF was responsible for the impaired OB differentiation induced by *Niban2* deficiency, we performed TF enrichment analysis with phenotype‐related DEGs (Figure 3i). *Runx2*, as the master transcription factor in OB differentiation,^[^ ^2^ ^]^ was enriched in our TF enrichment analysis and showed the lowest qvalve (Figure 3i).

**Figure 4:**
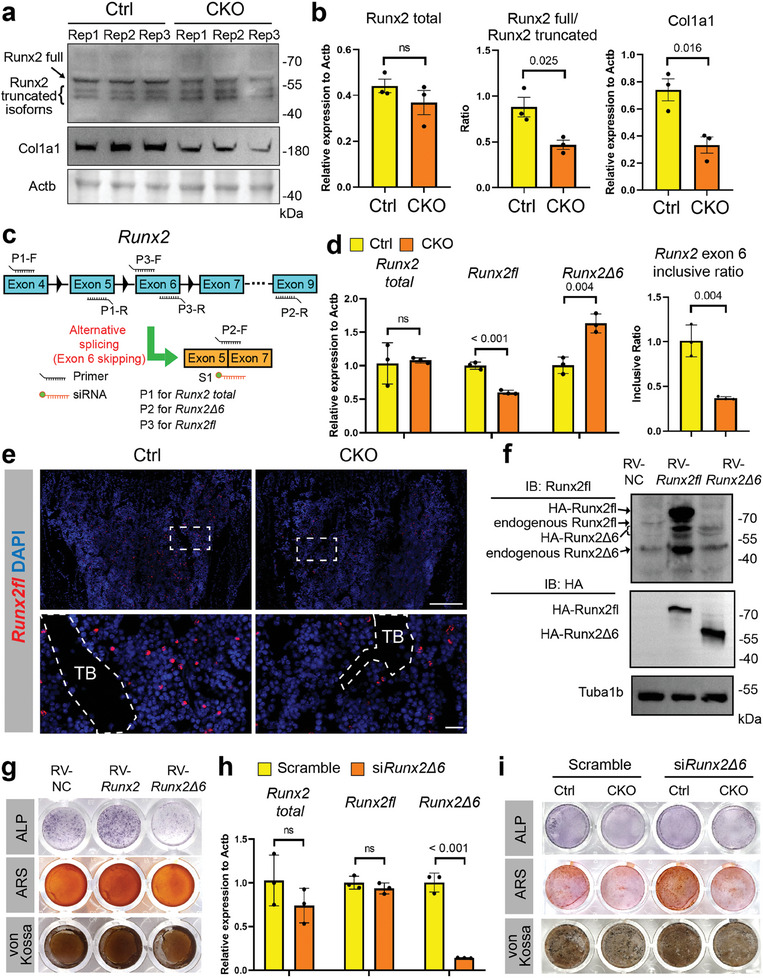
*Runx2 exon 6* is essential for osteoblast differentiation and regulated by Niban2. a) Western blot of Runx2 isoform and Col1a1 expression in the cranium of *Niban2* CKO mice and control mice. b) Quantitative analysis of the western blot of panel a. c) Schematic of primers and siRNA design for *Runx2* exon 6 AS. d) The results of quantitative RT‒PCR to detect *Runx2 total*, *Runx2fl*, *Runx2Δ6*, and *Runx2* exon 6 inclusive ratio in *Niban2* CKO pre‐OBs and the controls. e) RNA‐FISH of *Runx2fl* in *Niban2* CKO mice and control mice (scale bar: 200 µm; for magnification, scale bar: 20 µm; TB: trabecular bone). f) Western blot of Runx2 isoform and HA tag expression in *Runx2fl‐ and Runx2Δ6*‐overexpressing pre‐OBs and control cells. g) OB differentiation staining of *Runx2fl‐ and Runx2Δ6*‐overexpressing pre‐OBs and control cells, including ALP, ARS, and von Kossa staining. h) The results of quantitative RT‒PCR to detect *Runx2 total*, *Runx2fl*, and *Runx2Δ6* in scramble‐ or si*Runx2Δ6‐*transfected pre‐OBs. i) OB differentiation staining after scramble or si*Runx2Δ6* transfection in *Niban2* CKO pre‐OBs and the controls, including ALP, ARS, and von Kossa staining. Data are presented as the mean (SD), and individual data are indicated as points. *p* values were tested by unpaired Student's *t*‐test or one‐way ANOVA followed by Bonferroni's post hoc test. *p* values were presented if it < 0.05.

To further explore whether it was Runx2 that mainly contributed to Niban2‐related osteogenesis degeneration, we detected its expression in bone tissue of *Niban2* CKO mouse with its control. To our surprise, the *Runx2* expression levels displayed no significant difference (**Figure** **4**a). Consistently, Runx2 with largest molecular weight (>55 kDa, and estimated as Runx2 isoforms with full length^[^ ^8^ ^]^) were significantly downregulated, whereas Runx2 isoforms with smaller molecular weight (<55 kDa, various bands) were upregulated in the *Niban2* KO mice (Figure 4a,b). To note, there were no difference in the total Runx2 expression (all isoforms summed) between two genotypes (Figure 4b). While Col1a1, as one of the Runx2 downstream, was downregulated in *Niban2* CKO group (Figure 4b). In other words, different isoforms of Runx2 protein exhibited distinct expression patterns rather than difference in expression level in these two genotypes. Alternative splicing (AS) is known to regulate the expression of multiple isoforms of *Runx2* with distinct functions.^[^ ^8^ ^]^ Therefore, splicing event (SE) analysis on our RNA‐seq dataset was further performed by using rMATS.^[^ ^9^ ^]^ A significant increase in AS events was observed in the *Niban2*‐deficient pre‐OBs compared to that in control cells (Figure S5a, Supporting Information). In particular, CKO cells exhibited lower *Runx2* exon 6‐inclusive levels than control cells in rMATS analysis, which indicated a lower ratio of *Runx2* transcripts with exon 6 versus that without exon 6 transcripts (Figure S5b,c, Supporting Information). To verify the distinct expression levels of *Runx2* isoforms in the transcriptome, we generated different pairs of primers to detect specific *Runx2* transcripts (Figure 4c). Similar to the normalized expression level of *Runx2* in RNA‐seq (Figure 3b), quantitative RT‒PCR did not detect a significant difference in the expression level of *Runx2* with conservative region^[^ ^8^, ^10^ ^]^ (hereafter named *Runx2 total*) in the two types (Figure 4d). Notably, exon 6‐inclusive *Runx2* transcripts (hereafter named *Runx2fl*) significantly decreased, whereas exon 6‐exclusive *Runx2* transcripts (hereafter named *Runx2Δ6*) increased in CKO pre‐OBs (Figure 4d). The reduction of *Runx2fl* was verified in distal femoral trabecular regions of the *Niban2* CKO mice compared to that of control mice by in situ hybridization (RNA‐FISH) assay (Figure 4e). These observations suggest that Runx2 may be a potential target by which Niban2 promotes OB differentiation.

### 2.5 Runx2 Exon 6 is Essential for Osteoblast Differentiation

Then we performed further examinations to verify whether the distinct expression levels of *Runx2* AS transcripts attributed to its isoform expression levels in protein. Different Runx2 isoforms exhibit distinct functions in OB differentiation^[^ ^8^, ^10^ ^]^ *Runx2* exon 6 (NCBI RefSeq: NM_0 011 46038.3: 948–1121) encodes parts of the nuclear localization signal (NLS, PRRHRQKLD) of the Runt‐homology domain.^[^ ^11^ ^]^ We verified that the overexpression of the *Runx2Δ6* (full‐length except exon 6) isoform impeded OB differentiation, whereas *Runx2fl* (full‐length) promoted OB differentiation with quantitative analysis (Figure 4f,g and Figure S5d, Supporting Information). As to the possible reason why *Runx2Δ6* caused OB inhibition, we speculated that it might be caused by crosstalk or competition between Runx2Δ6 and endogenous Runx2, which needed to be further verified in subsequent studies. Notably, our overexpression backbone contains 3XHA and the hinge region, which resulted higher location of HA‐Runx2fl and Runx2fl then the endogenous products in western blots (Figure 4f). Interestingly, overexpression of *Runx2fl* increased endogenous Runx2 isoform expression, including *Runx2Δ6*, while overexpression of *Runx2Δ6* had little effect (Figure 4f). Most importantly, silencing *Runx2Δ6* (illustration of the design in Figure 4c) rescued the impaired OB differentiation with quantitative data due to *Niban2* deficiency (Figure 4h,i and Figure S5e, Supporting Information). Our results demonstrate that Niban2 promotes OB differentiation by regulating *Runx2* alternative splicing and decreasing exon 6‐exclusive Runx2 isoforms.

### 2.6 Hnrnpu is Essential for the Effect of Niban2 on Osteoblast Differentiation

**Figure 5:**
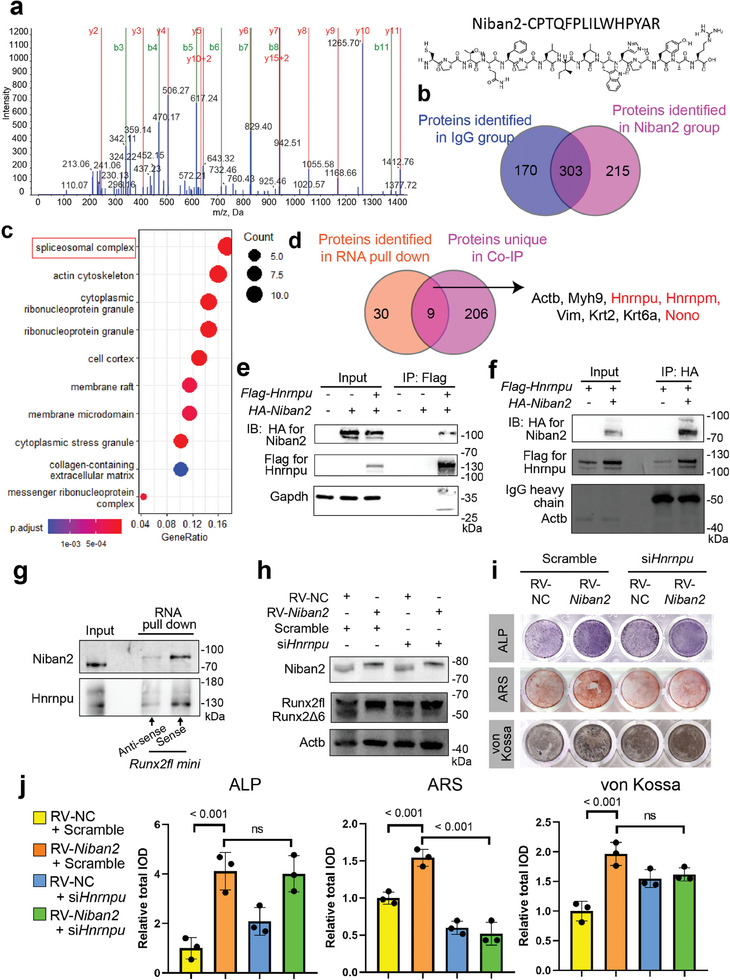
Niban2 interacts with the Hnrnpu‐cored spliceosome complex. a) Representative peak diagram of the Niban2 peptide in mass spectrometry (MS) analysis. b) Venn diagram of proteins identified by coimmunoprecipitation followed by MS in the Niban2 and IgG groups. c) Bubble plot of GO cellular component analysis of all identified proteins. d) Venn diagram of *Runx2* exon 6 binding proteins identified by RNA pulldown and Niban2 binding proteins identified by coimmunoprecipitation. RBPs are marked in red. e,f) Co‐IP was performed with anti‐Flag antibody for Flag‐Hnrnpu or anti‐HA antibody for HA‐Niban2. Immunoblotting (IB) was carried out to detect Niban2, Hnrnpu, Gapdh, or Actb. g) RNA pulldown was conducted with biotin‐labeled *Runx2fl mini* or scramble RNA, and IB was carried out to detect Niban2 and Hnrnpu. h) Western blot of Runx2 after scramble or si*Hnrnpu* transfection in *Niban2‐OE* MC3T3‐E1 and the controls. i) OB differentiation staining of *Niban2‐OE* MC3T3‐E1 and the controls after scramble or si*Hnrnpu* transfection, including ALP, ARS, and von Kossa staining. j) Quantitative analysis of the osteoblast differentiation staining of panel i. *p* values were tested by one‐way ANOVA followed by Bonferroni's post hoc test. *p* values were presented if it ≤ 0.05.

To explore how Niban2 participates in *Runx2* alternative splicing, we immunoprecipitated NIBAN2 and performed mass spectrometry (MS) to identify NIBAN2‐interacting proteins. The representative peak diagram of the unique Niban2 peptide was presented and demonstrated successful immunoprecipitation (IP) of Niban2 (**Figures** **5**a and S5f, Supporting Information). In total, 215 proteins were identified interacting with Niban2 (Figure 5b). As expected, varieties of RNA‐binding proteins were significantly enriched, and the spliceosome complex was the most enriched signature (Figure 5c). Particularly, multiple splicing factors, including Hnrnpu, Hnrnpk, Hnrnpm, Hnrnpdl, Nono, and Hnrnpr, were among the top 15 proteins (Figure S5g, Supporting Information). Then, to investigate which components may mediate *Runx2* alternative splicing due to *Niban2* deficiency, we constructed a *Runx2fl* reporter minigene for in vitro transcription (*Runx2* exon 6 with 150 bp of flanking intron for each side was transcribed, hereafter named *Runx2lf mini*) and RNA pulldown followed by mass spectrometry as previously described^[^ ^12^ ^]^ (Figure S6a,b, Supporting Information). We identified 9 proteins that overlapped in *Runx2fl mini* RNA pulldown and Niban2 Co‐IP, in which Hnrnpu, Hnrnpm, and Nono were RNA‐binding proteins in the spliceosomal complex^[^ ^13^ ^]^ (Figure 5f). Among the above three candidates, Hnrnpu was selected as the potential key protein due to its highest score and coverage in co‐IP MS (Figure S5g, Supporting Information). The interaction of Hnrnpu with Niban2 was further verified by Co‐IP followed by western blot (Figure 5e,f, Supporting Information), and the interaction of *Runx2lf mini* with Hnrnpu and Niban2 were further verified by RNA pulldown followed by western blot (Figure 5g). These results demonstrate that Niban2, Hnrnpu, and *Runx2* transcripts (pre‐mRNA) form a spliceosomal complex. Moreover, *Hnrnpu* knockdown in the pre‐OB cell line offset the effect of *Niban2* overexpression on the expression pattern of the Runx2 isoforms (Figure 5h). This phenotype demonstrated the importance of Runx2Δ6 in the regulation of OB differentiation. Most importantly, downregulation of *Hnrnpu* abrogated the *Niban2* overexpression‐enhanced OB differentiation with quantitative analysis (Figure 5i,j and Figure S6c, Supporting Information). These observations indicate that Hnrnpu mediates Niban2 function in OB differentiation.

### 2.7 Niban2 Alters Components of the Hnrnpu‐Cored Spliceosome Complex

**Figure 6:**
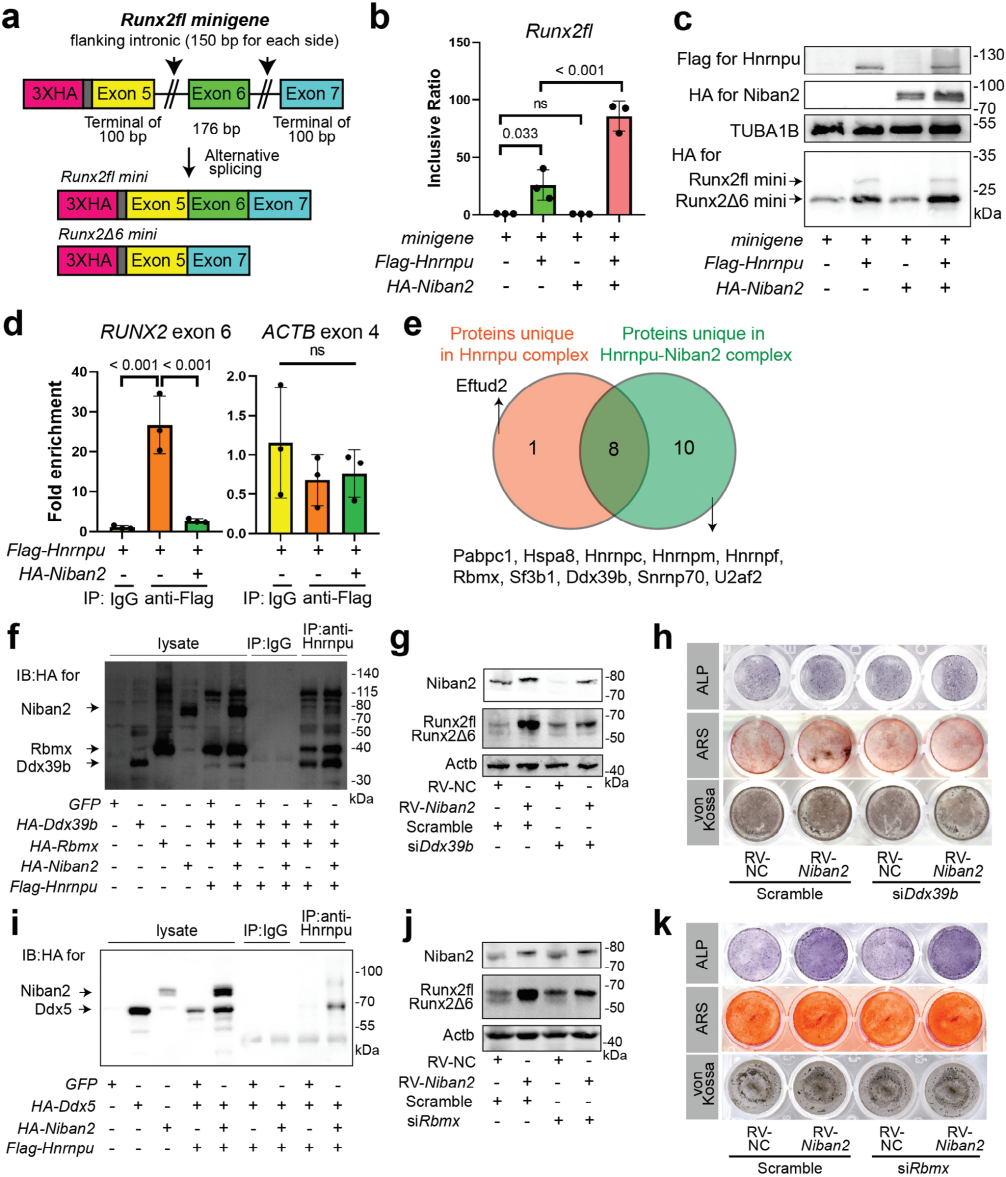
Alteration of the Hnrnpu‐cored spliceosome complex by Niban2 contributes to *Runx2* alternative splicing and OB differentiation. a) Schematic of the *Runx2fl* minigene reporter. b) The results of quantitative RT‒PCR to detect *Runx2 exon 6* inclusive ratio in cells with *Niban2* and/or *Hnrnpu* overexpression and the control cells. c) Representative western blot to detect *Runx2fl mini* and *Runx2Δ6 mini* in HEK293T with *Niban2* and/or *Hnrnpu* overexpression and the control cells. d) The results of quantitative RT‒PCR to detect *RUNX2* exon 6 and *ACTB* exon 4 in RBP immunoprecipitations by Hnrnpu with or without Niban2 overexpression. HEK293T cells were first transfected with plasmids expressing HA‐labeled spliceosome complex components. Co‐IP was then performed with anti‐Hnrnpu antibody or preimmune IgG. e) Venn diagram of MS‐identified Hnrnpu binding proteins in *Niban2‐OE* cells and control cells. f) IB was carried out to detect Niban2 and the other spliceosome complex components, Rbmx and Ddx39b. g) Western blot of Runx2 after scramble or si*Ddx39b* transfection in *Niban2‐OE* MC3T3‐E1 and control cells. h) OB differentiation staining of *Niban2‐OE* MC3T3‐E1 and the controls after scramble or si*Ddx39b* transfection, including ALP, ARS, and von Kossa staining. i) IB was carried out to detect Niban2 and the other spliceosome complex component, Ddx5. j) Western blot of Runx2 after scramble or si*Rbmx* transfection in *Niban2‐OE* MC3T3‐E1 and control cells. k) OB differentiation staining of *Niban2‐OE* MC3T3‐E1 and the controls after scramble or si*Rbmx* transfection, including ALP, ARS, and von Kossa staining. Data are presented as the mean (SD) and individual data are indicated as points. *p* values were tested by one‐way ANOVA followed by Bonferroni's post hoc test. *p* values were presented if it < 0.05.

To test whether *Hnrnpu* was required for *Niban2*‐mediated alternative splicing of *Runx2*, we used the *Runx2fl* reporter minigene system in HEK293T cells (**Figure** **6**a). Different isoforms with or without *Runx2* exon 6 (hereafter named *Runx2fl mini* and *Runx2Δ6 mini*) were measured by quantitative RT‒PCR or Western blots for HA (Figure 6b,c). *Flag‐Hnrnpu* overexpression significantly induced *Runx2fl mini* transcription (Figure 6b,c). Moreover, *HA‐Niban2* overexpression further enhanced the Runx2fl isoforms under *Flag‐Hnrnpu* overexpression, while HA‐Niban2 alone did not increase *Runx2fl* transcription (Figure 6b,c).

To further dissect the molecular basis by which Niban2 regulates *Runx2* AS through Hnrnpu, we performed RIP and showed a significant reduction in Flag‐Hnrnpu binding to the *RUNX2* transcripts with exon 6 (Figure 6d). Hnrnpu belongs to the hnRNP family, which is a common component of the spliceosome and regulates AS by binding to an exon splice silencer (ESS).^[^ ^14^ ^]^ Recognition of ESS by hnRNPs is a signal for exon skipping.^[^ ^14^ ^]^ Therefore, the decreased binding of Hnrnpu to *Runx2* exon 6 caused by Niban2 was highly related to the AS process. Indeed, overexpression of *HA‐Niban2* affected the composition of the spliceosome complex, including the absence of Eftud2 and the presence of 10 other spliceosome components (Figure 6e). Western blot analysis verified that *HA‐Niban2* overexpression increased Ddx5, Rbmx, and Ddx39b binding to Hnrnpu (Figure 6f–i), and no remaining lysate was detected in each IP group (Figure S6 d, Supporting Information). Notably, we also barely observed binding between Hnrnpu and Hnrnpc, and the absence of Eftud2 in MS could not be repeated by western blotting (Figure S6 e, Supporting Information). Finally, we downregulated *Ddx39b* in *Niban2*‐OE cells (Figure S6 f, Supporting Information). *Ddx39b* knockdown in the pre‐OB cell line abrogated the downregulation of *Runx2Δ6* and upregulation of *Runx2fl* attributed to *Niban2* overexpression (Figure 6g). ALP staining revealed that *Ddx39b* knockdown marginally affected early OB differentiation (Figure 6h and Figure S6 g, Supporting Information). However, ARS and von Kossa staining revealed that *Ddx39b* deficiency offset the enhanced mineralization induced by Niban2 (Figure 6h and Figure S6 g, Supporting Information). Solely *Rbmx* deficiency in pre‐OB cell line already downregulated *Runx2Δ6* and upregulated *Runx2fl*, which barely influenced Niban2‐induced *Runx2* AS switch (Figure 6j). Independent regulation of *Niban2*‐OE and *Rbmx* deficiency were also observed in OB differentiation staining (Figure 6k and Figure S7a,b, Supporting Information). Effect of spliceosome composition on *Runx2* AS, especially on *Runx2Δ6*, was further detected in pre‐OB cell line with *Runx2fl* reporter minigene system (Figure S7c, Supporting Information). Knockdown of *Hnrnpu* or *Ddx39b*, respectively, onset the downregulation of *Runx2Δ6* caused by *Niban2*, of which knockdown of *Rbmx* failed (Figure S7c, Supporting Information).

Taken together, our results demonstrate that Niban2 binds to Hnrnpu and subsequently alters the composition of the Hnrnpu‐cored spliceosome complex, leading to increased *Runx2* exon 6‐inclusive transcripts and promoting OB differentiation.

### 2.8 NIBAN2‐Regulated RUNX2 Alternative Splicing is Tightly Associated with Osteoporosis

**Figure 7:**
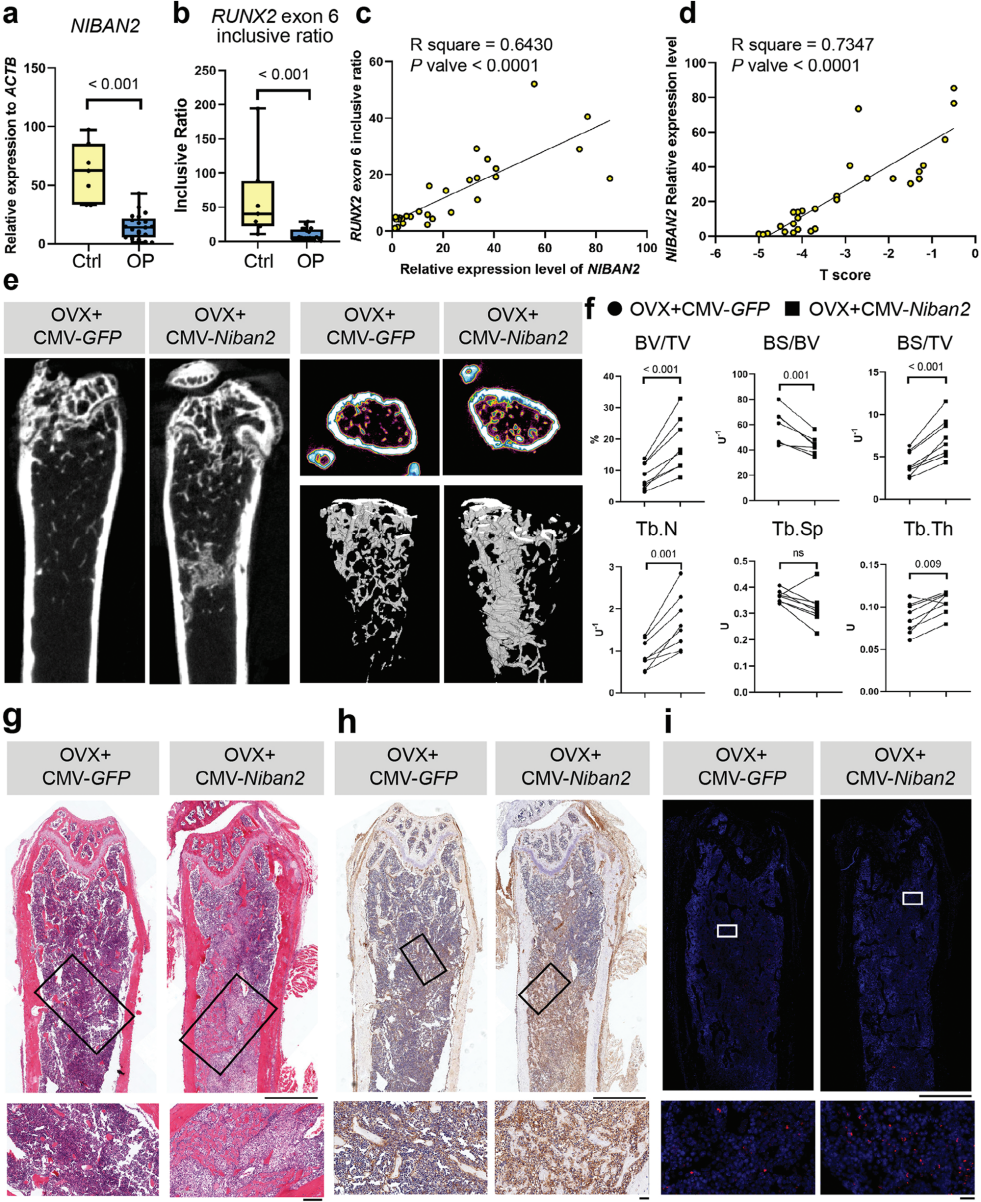
The clinical relevance and therapeutic effect of NIBAN2‐regulated RUNX2 alternative splicing on osteoporosis. Relative expression of a) *NIBAN2* to *ACTB* and b) *RUNX2* exon 6 inclusive ratio in osteoporosis patients and controls (n = 8 in the control group, n = 20 in the osteoporosis group). c) Linear regression of the relative expression level of *NIBAN2* and *RUNX2* exon 6 inclusive ratio in enrolled patients d) Linear regression of the relative expression level of *NIBAN2* and T score in enrolled patients. e) Coronal section, cross section, and reconstruction images of representative *Niban2‐OE* femurs and control femurs by µCT. f) Quantitative analysis of the µCT results of e. g) H&E staining of *Niban2* CKO mice and control mice (scale bar: 1 mm; for magnification, scale bar: 200 µm). Representative images for 3 independent samples. h) Immunohistochemistry staining of Niban2 in *Niban2‐OE* femurs and control femurs (scale bar: 1 mm; for magnification, scale bar: 50 µm). Representative images for 3 independent samples. i) RNA‐FISH of *Runx2* exon 6 in *Niban2‐OE* femurs and control femurs (scale bar: 1 mm; for magnification, scale bar: 20 µm). Representative images for 3 independent samples. Data are presented as the mean (SD), except min to max bar and quarters in boxplots, and individual data are indicated as points. *p* values were tested by paired or unpaired Student's *t*‐test. *p* values were presented if it < 0.05.

To address the clinical relevance of our findings, we collected bone tissues from osteoporosis patients and non‐osteoporosis control and measured *RUNX2* alternative splicing and the expression level of *NIBAN2*. Cancellous bone samples were collected from osteoporosis and non‐osteoporosis patients at their orthopedic surgery for vertebral fractures. A significant decrease in osteoblast activity and a slight increase in osteoclastic activity were observed in our enrolled samples, which was consistent with the typical pathological phenotype of senile osteoporosis (Figure S7d,e, Supporting Information).^[^ ^15^ ^]^ Bone tissues from the osteoporosis patients exhibited significantly lower expression levels of *NIBAN2* and *RUNX2* exon 6‐inclusive ratio than those from the control non‐osteoporosis patients (**Figure** **7**a,b). Notably, lower *RUNX2* exon 6‐inclusive ratio in osteoporosis patients was attributed lower level of *RUNX2FL*, rather than elevated level of *RUNX2Δ6* (Figure S7 f,g, Supporting Information). The *NIBAN2* expression level was positively correlated with the *RUNX2* exon 6‐inclusive ratio in osteoporosis patients (Figure 7c). More importantly, the T score (representing bone mineral density, measured by dual energy X‐ray absorptiometry) was positively correlated with the relative *NIBAN2* expression level (Figure 7d). These observations demonstrate that *RUNX2* AS and NIBAN2 expression are tightly correlated with osteoporosis and may serve as biomarkers in the clinic.

### 2.9 Niban2 Rescues Ovariectomy‐Induced Osteoporosis in Mice

To examine the potential therapeutic effect of Niban2 in osteoporosis in vivo, we overexpressed *Niban2* by intramedullary transduction in ovariectomized (OVX) mouse femurs.^[^ ^16^ ^]^ Bone loss was confirmed in the OVX mice without intramedullary transduction compared to the sham mice (Figure S8 a,b, Supporting Information). In parallel, each femur of the same mice was transduced with the CMV‐Niban2 overexpression vector and the CMV‐*GFP* control vector (hereafter named the *Niban2* OE femur and the control femur). As expected, the control OVX mice exhibited less trabecular bone in µCT 3D reconstruction images than the Niban2 OE mice, while significant reinforced bone formation was found in the marrow cavity of the *Niban2* OE femurs (Figure 7e). Further quantitative analysis demonstrated that BV/TV, Tb. N, and trabecular thickness (Tb. Th) significantly increased as BS/BV significantly decreased, suggesting that the increase in bone regeneration during osteoporotic progression was attributed to CMV‐*Niban2* injection (Figure 7f). H&E staining illustrated that neonatal high‐density structures appearing on µCT in the *Niban2* OE femurs had typical bone histological characteristics (Figure 7g). The immunohistochemistry of Niban2 verified significantly higher expression levels of Niban2 in the *Niban2* OE femurs (Figure 7h and Figure S8c, Supporting Information). Meanwhile, a significant increase of osteogenesis collagen, Bglap, was observed in the *Niban2* OE femurs (Figure S8d,e, Supporting Information), with no significant alteration of osteoclast activity (Figure S8f,g, Supporting Information). Finally, RNA‐FISH confirmed that there was an increase in *Runx2* exon 6‐included transcripts in *Niban2* OE femurs (Figure 7i). In summary, Niban2 overexpression regulates *Runx2* AS and rescues bone loss in OBs during osteoporosis in vivo.

## 3 Discussion

Osteoporosis causes a major economic burden worldwide.^[^ ^17^ ^]^ Enhancing OB differentiation to antagonize excessive bone loss or defects in bone formation has been proposed as a novel strategy.^[^ ^18^ ^]^ Although multiple factors promote OB differentiation, especially transcription factors in the nucleus, few of them have been targeted for therapy.^[^ ^7^, ^19^ ^]^ In this research, NIBAN2 was identified as a novel factor promoting OB differentiation through regulation of *RUNX2* alternative splicing. This research provides a potential strategy for osteoporosis therapy, which is difficult to target before.

Our study reveals a novel function of NIBAN2 in OB differentiation and osteoporosis. Previous studies have shown that NIBAN2 serves as an unfavorable prognostic factor in multiple cancers.^[^ ^6^, ^20^ ^]^ In addition, NIBAN2 is essential for wound healing^[^ ^21^ ^]^ and ameliorates myocardial ischemia‒reperfusion injury.^[^ ^6b^ ^]^ Through integrative analysis of multiple databases, NIBAN2 was identified as the most promising osteogenic gene downregulated in osteoporosis. The function of NIBAN2 in OB differentiation was confirmed in vitro and in vivo. Moreover, multiple skeletal abnormalities and deficiencies, including loss of trabeculae, insufficient mineralization, and sluggish bone turnover, were confirmed in CKO mice, which was attributed to a reduction in OB function due to delayed OB differentiation. Notably, reduced OC was also observed in CKO mice rather than in vitro (Figure S4h–k, Supporting Information). These results indicated that *Niban2* deficiency in the OB lineage implied osteoblast‐osteoclast communication. Although the above observations could be explained by reduced OB secretory functions caused by decreased OB differentiation, further studies should be conducted to explore whether a neglected mechanism exists in cell‒cell interactions. Overall, our research revealed the essential role of NIBAN2 in bone health. Furthermore, NIBAN2 downregulation was tightly associated with osteoporosis, and NIBAN2 overexpression rescued bone loss in OVX‐induced osteoporosis. These results demonstrate that NIBAN2 plays an important role in OB differentiation and osteoporosis.

Our study also unveils novel mechanisms by which NIBAN2 regulates OB differentiation. Previous studies have shown that NIBAN2 functions through binding of its C‐terminal flexible region to other proteins,^[^ ^22^ ^]^ such as Ras,^[^ ^20a^ ^]^ large tumor suppressor kinase 1 (LATS1),^[^ ^23^ ^]^ and kelch‐like ECH associated protein 1 (KEAP1),^[^ ^20c^ ^]^ resulting in activation or suppression of its ligands. In this research, Niban2 bound to the core component of the spliceosome complex Hnrnpu and altered the composition of the spliceosome, which enhanced Hnrnpu recognition of *Runx2fl* and promoted AS of *Runx2* transcripts retaining exon 6. More experimental evidence is needed to confirm whether the PH domain in the N‐terminus or flexible region in the C‐terminus of NIBAN2 directly interacts with HNRNPU. Moreover, whether phosphorylation of NIBAN2 (phosphorylation sites at Tyr593, Ser641, Ser646, and so on)^[^ ^20^, ^22^ ^]^ affects its binding to HNRNPU requires further confirmation. RUNX2 is known to has two opposing stage‐specific functions. It promotes OB differentiation in initial stages whereas inhibits late differentiation of mature OBs.^[^ ^24^ ^]^ This conclusion was mainly based on results of transgenic model with exogenous RUNX2 overexpressing in collagen‐expressing OBs.^[^ ^24^ ^]^ In this research, NIBAN2 was deleted in pre‐OBs via Bglap‐Cre with impaired OB differentiation in vivo and in vitro attributing to increasing *RUNX2* exon 6 AS. This demonstrates a novel AS regulation of RUNX2 on OB differentiation, which differs from exogenous overexpression. *RUNX2* exon 6 encodes part of the NLS (PRRHRQKLD) of the Runt‐homology domain that causes the accumulation of RUNX2 isoform in the cytoplasm.^[^ ^8^, ^11^, ^25^ ^]^ Thus, exon 6 skipping determines whether RUNX2 can activate downstream osteogenic genes. The mutation of RUNX2 gene targeting Runt domain and its NLS were reported to associated with subcellular localization of the mutant protein and cleidocranial dysplasia, an inherited bone disorder disease.^[^ ^11^, ^26^ ^]^ However, AS transcripts of *RUNX2* lacking exon 6 (*Runx2Δ6*) were barely reported and paid little attention on its role in bone biology.^[^ ^8^, ^11^, ^27^ ^]^ Thus, our research unraveled a potential mechanism about NIBAN2 on *RUNX2* homeostasis.

In fact, HNRNPU regulates U2 snRNP maturation, which governs *Runx2* exon skipping AS,^[^ ^27^, ^28^ ^]^ and AS contributes to the regulation of OB differentiation.^[^ ^8^, ^10^, ^27^ ^]^ In this research, the *Runx2fl* minigene reporter system was constructed to detect *Runx2* exon 6 AS. The distinct AS pattern of *Runx2 mini* was detected between pre‐OB cell and non‐OB lineage cell line (HEK293T). Pre‐*Runx2 mini* was mainly processed to *Runx2fl* in pre‐OB cell line (Figure S7c, Supporting Information), of which was processed to *Runx2Δ6* in HEK293T (Figure 6c), and this emphasizes the specialty of *Runx2* AS pattern in bone. It is possible that NIBAN2 directly regulates downstream transcription, although NIBAN2 was not found to have a DNA or RNA binding motif in the current structural study.^[^ ^22^ ^]^ Indeed, transcription and AS are continuous processes in the regulation of gene expression.^[^ ^29^ ^]^ Thus, further mechanistic research should be performed to explore the details of posttranscriptional regulation by NIBAN2 in bone homeostasis, such as structural studies. Our study highlights the importance of AS in OB differentiation and identifies NIBAN2 as a novel regulator of AS.

Our study suggests that NIBAN2 may be a potential target for anabolic therapy of osteoporosis. Currently, pharmacologic agents for the treatment of osteoporosis can be classified as either antiresorptive or anabolic.^[^ ^19^ ^]^ However, there are side effects of antiresorptive drugs, particularly bisphosphonates, and no clear evidence supports long‐term efficacy.^[^ ^18^ ^]^ Nonetheless, current anabolic agents are mostly short‐term and have other side effects.^[^ ^30^ ^]^ Thus, there is an urgent need to develop novel anabolic strategies with prolonged anabolic effects on bone. However, OB differentiation is tightly controlled by key transcription factors that are known to be difficult to target. RUNX2 is the master osteogenic transcription factor controlling the transcription of other downstream osteogenic genes and skeletal collagens. Notably, few mediator complexes have been reported to regulate RUNX2 function.^[^ ^31^ ^]^ NIBAN2 may provide alternative ways to target transcription factors such as RUNX2. Targeting AS via NIBAN2 to regulate key transcription factors is a novel strategy to enhance bone anabolism with a promising future. Thus, NIBAN2 may be used to design pharmacological intervention strategies since it harbors numerous posttranslational modification sites and motifs that serve as candidates for pharmacological intervention.^[^ ^22^ ^]^ Further dissecting the interface of NIBAN2 interacting with other components might provide detailed information to develop compounds for mimicking NIBAN2. In this way, alternative splicing of *RUNX2* retaining exon 6 may be enhanced to promote OB differentiation with the presentation of NIBAN2.

There are still some limitations in this study. One of them is the lack of single‐cell evidence to prove the association between the RUNX2 AS pattern and OB differentiation, and degenerative bone diseases. This is attributed to the inadequacy of current commercial single‐cell sequencing in alternative splicing analysis, and will be solved via full‐length single‐cell sequencing in future. With single‐cell AS cues, the fate determination of OB differentiation and the pathogenesis of osteoporosis will be further clarified.

## 4 Conclusion

In conclusion, our study identified NIBAN2 as a new factor that promotes OB differentiation by regulating the alternative splicing of *RUNX2*. Mechanistically, NIBAN2 interacted with the HNRNPU‐cored spliceosome complex and altered its components to regulate the alternative splicing of *RUNX2*, which ultimately caused an increase in functional RUNX2 (nuclear localization sequence complete) but a decrease in dysfunctional RUNX2 (exon 6 exclusive) to reinforce osteoblast differentiation. Most importantly, NIBAN2 correlation to RUNX2 alternative splicing and bone loss was verified in osteoporosis patients. NIBAN2 rescued bone loss in postmenopausal osteoporosis model. Thus, our research identifies NIBAN2‐regulated RUNX2 alternative splicing as a potential mechanism of osteoblast differentiation that may present strategies for antagonizing osteoporosis.

## 5 Experimental Section

### Study Approval

This work had complied with all relevant ethical regulations for clinical samples and animal research. Cancellous bone samples were collected from osteoporosis and nonosteoporosis patients at their orthopedic surgery for vertebral fracture. The human study of this research was conducted in accordance with the principles expressed in the *Declaration of Helsinki* and was approved by the ethical committee of the Medical Ethical Committee of Zhongnan Hospital of Wuhan University (approval number: 2022058K). Written informed consent was obtained from each enrolled patient. All animal experiments were performed in the Central of Experimental Animal Zhongnan Hospital of Wuhan University. The animal experiments were conducted according to the protocol (approval number: ZN2021176) authorized by the Experimental Animal Welfare Ethics Committee, Zhongnan Hospital of Wuhan University.

### Mouse Lines and Animal Experiments Design

*Niban2^flox/flox^* mice (Cyagen Biosciences, China, strain ID: CKOCMP‐227737‐Niban2‐B6N‐VA) were crossed with the *Bglap^Cre^* strain (Cyagen Biosciences, China, strain ID: C001025). For *Bglap^Cre^*; *Niban2^flox/flox^* mice, littermate *Niban2^flox/flox^* mice served as controls. All mice analyzed were maintained on the C57BL/6 background. Animal experimental unit in this research referred to a single animal. No specific criteria, except age and gender, were set to inclusive or exclusive mice. The order in which the animals were tested (like µCT) during the experiment was randomized. The animal keepers, examiner, and data analyst were not aware of the group allocation. All the animal experiments were performed in Division of Laboratory Animal Services, Zhongnan Hospital of Wuhan University. All mice were housed individually in standard cages with a 12:12 h light/dark cycle and room temperature maintained at 21 ± 1 °C.

### Isolation and Culture of Pre‐Osteoblasts

Primary pre‐OBs were isolated from 3 weeks male mice according to previously reported protocols and modified in detail.^[^ ^32^ ^]^ *Bglap^Cre^*; *Niban2^flox/flox^* mice, *Niban2^flox/flox^* mice, and C57BL/6 mice were sacrificed for the generation of pre‐OBs in each genotype. *Niban2* knockout in *Bglap^Cre^*; *Niban2^flox/flox^* pre‐OBs was induced via OB differentiation. The induction time was 8 days following our in vitro assay for knockout efficiency (Figure 3a). The detailed procedures of cell isolation, osteoblast differentiation, and additional information regarding cell lines used in this paper can be found in *Supplementary Material*, *Supplementary Methods*.

### Microcomputed Tomography Analysis

High‐resolution micro‐CT imaging system (Bruker, USA, SkyScan 1276) was applied in this research according to the assessment guidelines.^[^ ^33^ ^]^ Each femur was scanned separately at 55 kV and 200 µA using a 0.25‐mm aluminum filter to obtain an isometric resolution of 6 µm. NRecon (Bruker, USA) was used to reconstruct the image, and CTAn (Bruker, USA) was used for quantitative analysis.

### Histology Analysis

Femurs were fixed in 4% paraformaldehyde (Servicebio, China, G1101) for 48 h at 4 °C (except for frozen sections). For solid tissue sectioning, samples were embedded with polymethyl methacrylate (PMMA) and then cut into sections (4 µm thickness). Calcium deposits in the bone tissue were visualized by von Kossa staining using 4% silver nitrate (Servicebio, China, G1043) followed by hematoxylin‐eosin (H&E; Servicebio, China, G1005) counterstaining. For paraffin sectioning, femurs were first decalcified with EDTA solutions (Servicebio, China, G1105) at 4 °C. Then, sections (8 µm thickness) were prepared and stained with H&E and Goldner trichrome (Servicebio, China, G1064). For frozen sectioning, the time of fixation was shortened to 6 h, and decalcification was performed within 48 h by EDTA solutions with constant agitation as previously described.^[^ ^34^ ^]^ The additional information for immunohistochemistry, immunofluorescence, and other materials in this paper can be found in Supplementary Material, *Supplementary Methods*.

### Transfection of siRNA, Plasmid, Lentivirus and Retrovirus in vitro

A RiboFECT CP Transfection Kit (RiboBio, China, R10035.7) was used following the manufacturer's instructions for siRNA and plasmid transfection. The siRNA oligos used in this research were provided in Table S1, Supporting Information. An overexpression plasmid for *Niban2, Ddx5, Rbmx*, and *Ddx39b* was constructed by inserting their cDNA clones into pHAGE with 3×HA at the N‐terminus. The overexpression plasmid for *Hnrnpu* and *Niban2* with 3×FLAG at the C‐terminus was purchased from GeneChem (China), and the overexpression plasmid for *Niban2* was enveloped in lentivirus. For retroviral transfections of pre‐OBs, cDNA for *Runx2fl* and *Runx2Δ6* was first synthesized by Tsingke Biotechnology (China) and cloned into pMSCV. The plasmids pv78c and overexpression plasmids were cotransfected into HEK293T cells to produce retrovirus. Lentivirus and retroviral transfections were performed in the presence of 5 µg mL^−1^ polybrene (MOI = 20).

### Ovariectomy Model and In Vivo Transfection

Ovariectomy (OVX) was performed following a previous protocol.^[^ ^35^ ^]^ The same surgical procedure was followed for sham operations, but the ovaries were left intact. Two weeks after the operation, mice were processed for in vivo transfection of plasmids with Entranster‐in vivo (Engreen Biosystem, China, 18668‐11‐2). Each transfection complex contained 10 µg (diluted in 10 µL ddH_2_O) plasmid and 10 µL of transfection reagent. An intramedullary injection of femur was conducted at 2 weeks and 6 weeks after the surgery to perform tropical transfection in vivo. The intramedullary injection was processed as previously described.^[^ ^36^ ^]^ Matching groups were constructed by *Niban2*‐OE plasmid transfection in the left femur and CMV‐*GFP* transfection in the right femur for the same OVX mice.

### Quantitative RT‒PCR Analysis

Total RNA was extracted from cell samples using TRIzol (Thermo Fisher, USA, 15 596 026) following the instructions of the manufacturer. An aliquot of 500 ng of total RNA was reverse‐transcribed into cDNA with a reverse transcriptase kit (Vazyme, China, R223). Quantitative PCR was performed using a SYBR Green mixture (Vazyme, China, Q311) and a Monad Real‐Time PCR instrument (Monad, China, q225) or a Bio‐Rad Real‐Time PCR instrument (Bio‐Rad, USA, CFX384). The primers used for specific transcripts are listed in the Table S1, Supporting Information.

### Coimmunoprecipitation and Western Blot Analysis

Cell samples were collected and lysed in ice‐cold cell lysis buffer for Western blots and IP (Beyotime, China, P0013) containing both a protease inhibitor cocktail (MedChemExpress, China, HY‐K0010) and a phosphatase inhibitor cocktail (MedChemExpress, China, HY‐K0023). Cell lysates (1%) were preserved as inputs. Antibodies conjugated to magnetic beads against FLAG or HA (MedChemExpress, China, HY‐K0201 or HY‐K0207) and protein A‐G magnetic beads (MedChemExpress, China, HY‐K0202) were used to perform immunoprecipitation. Coimmunoprecipitated proteins were identified by mass spectrometry and verified by western blots. Western blotting was performed as previously described^[^ ^37^ ^]^ with primary antibodies and horseradish peroxidase‐linked secondary antibody (Cell Signaling Technology, USA, 7074). Images were acquired with an enhanced chemiluminescent imaging system (Tanon, China) without gamma adjustment in default parameters. The additional information for uncropped western blots, antibodies, and other materials can be found in *Supplementary Material*.

### Medical Illustrations

Figures in this research contain medical illustrations from SMART Servier Medical Art, reproduced with permission, licensed under a Creative Commons Attribution 4.0 unported license (https://creativecommons.org/licenses/by/4.0/).

### Statistics

Quantitative data are presented as the mean ± SEM, with P values of less than 0.05 considered significant. Parametric data were analyzed using the appropriate Student's t test when 2 groups were compared or a one‐way ANOVA when more than 2 groups were compared followed by Bonferroni multiple comparisons post hoc test as indicated in the figure legends. All statistical tests were performed using Prism 8.0 software (USA). Each experiment was performed at least three times independently.

### Data Availability

The RNA sequencing data have been deposited in the SRA under accession PRJNA899996. Correspondence and requests for materials or data should be addressed to RX. Wei, L. Cai, or Z. Huang. Other detailed information on the materials and software used in this paper is provided in *Supplementary Material*.

### Ethics Approval and Patient Consent Statement

This work had complied with all relevant ethical regulations for clinical samples and animal research. Cancellous bone samples were collected from osteoporosis and nonosteoporosis patients at their orthopedic surgery for vertebral fracture. The human study of this research was conducted in accordance with the principles expressed in the Declaration of Helsinki and was approved by the ethical committee of the Medical Ethical Committee of Zhongnan Hospital of Wuhan University (approval number: 2022058K). Written informed consent was obtained from each enrolled patient. All animal experiments were performed in the Central of Experimental Animal Zhongnan Hospital of Wuhan University. The animal experiments were conducted according to the protocol (approval number: ZN2021176) authorized by the Experimental Animal Welfare Ethics Committee, Zhongnan Hospital of Wuhan University

## Conflict of Interest

The authors declare no conflict of interest.

## Supporting information



Supporting Information

## Data Availability

The data that support the findings of this study are available from the corresponding author upon reasonable request.
