# Supplementary material for: Identification of NIBAN2‐Regulated RUNX2 Alternative Splicing Presents Novel Strategies for Antagonizing Osteoporosis
Source: Adv Sci (Weinh). 2025 Mar 7;12(17):2416536. doi: 10.1002/advs.202416536 (PMC12061305; doi:10.1002/advs.202416536)
Supplement: Supplementary file 1 — Supporting Information [file ADVS-12-2416536-s001.docx]

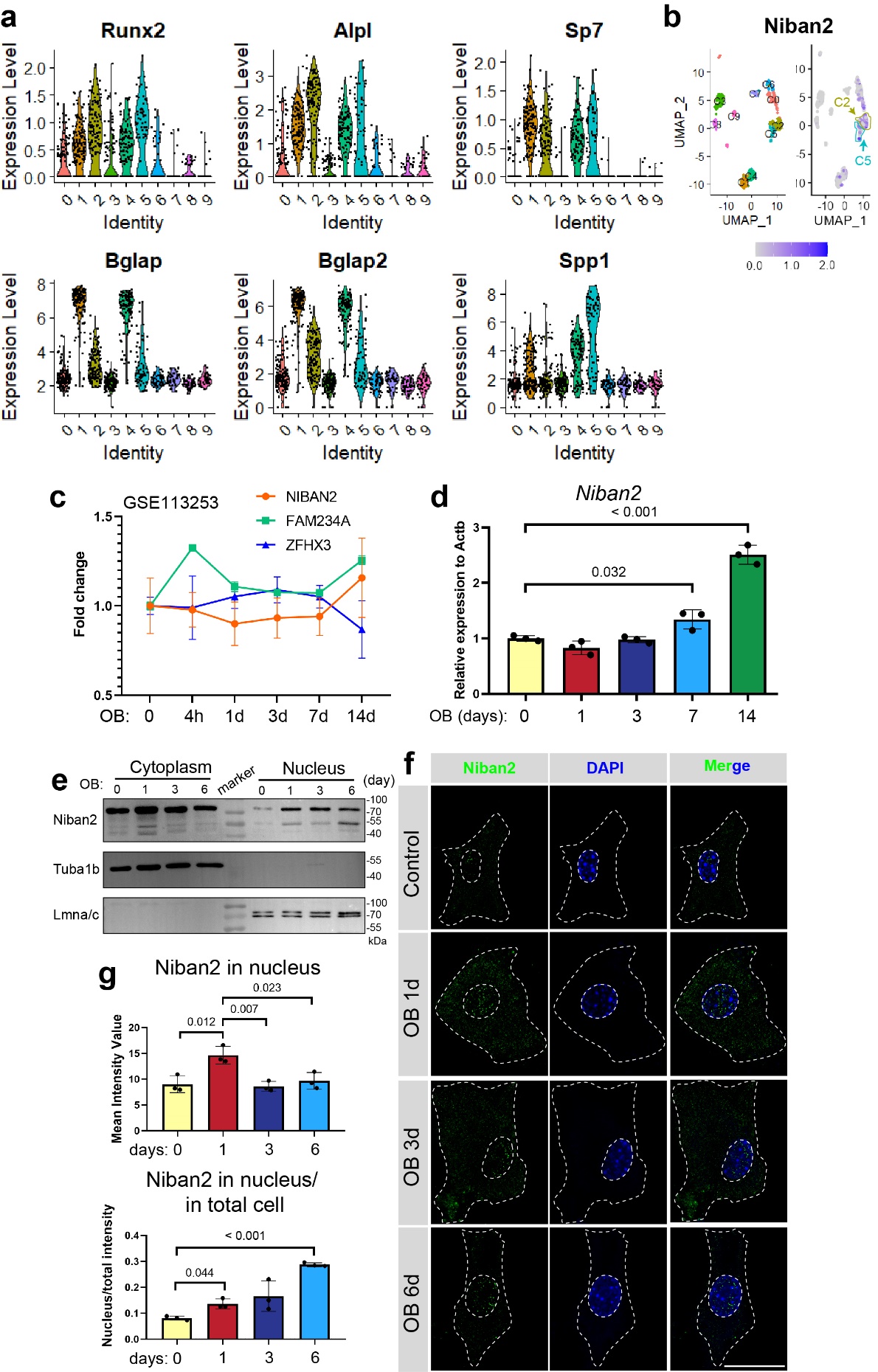
**Figure S1.** **Niban2 is correlated with OB differentiation and osteoporosis**

(a) Expression levels of OB function-related genes in each OB cluster in Figure 1a. (b) Feature blot of Niban2 in each OB cluster in Figure 1a. (c) *ZFHX3*, *FAM234A*, and *NIBAN2* mRNA foldchanges during OBs differentiation in dataset GSE. (d) Relative expression of *Niban2* to *Actb* during OB differentiation. (e) Western blot of Niban2 expression levels in the cytoplasm or nucleus during OB differentiation. Representative images for 3 independent repeats. (f) Immunofluorescence of Niban2 expression during OB differentiation (scale bar: 25 μm). Representative images for 3 independent samples. (g) Quantitative analysis of immunofluorescence in panel c with Niban2 expression levels in the cytoplasm or nucleus during OB differentiation. Data are presented as the mean (SD), and individual data are indicated as points. *P* values were tested by unpaired Student’s t test or one-way ANOVA followed by Bonferroni’s post hoc test. *P* values were presented if it < 0.05.


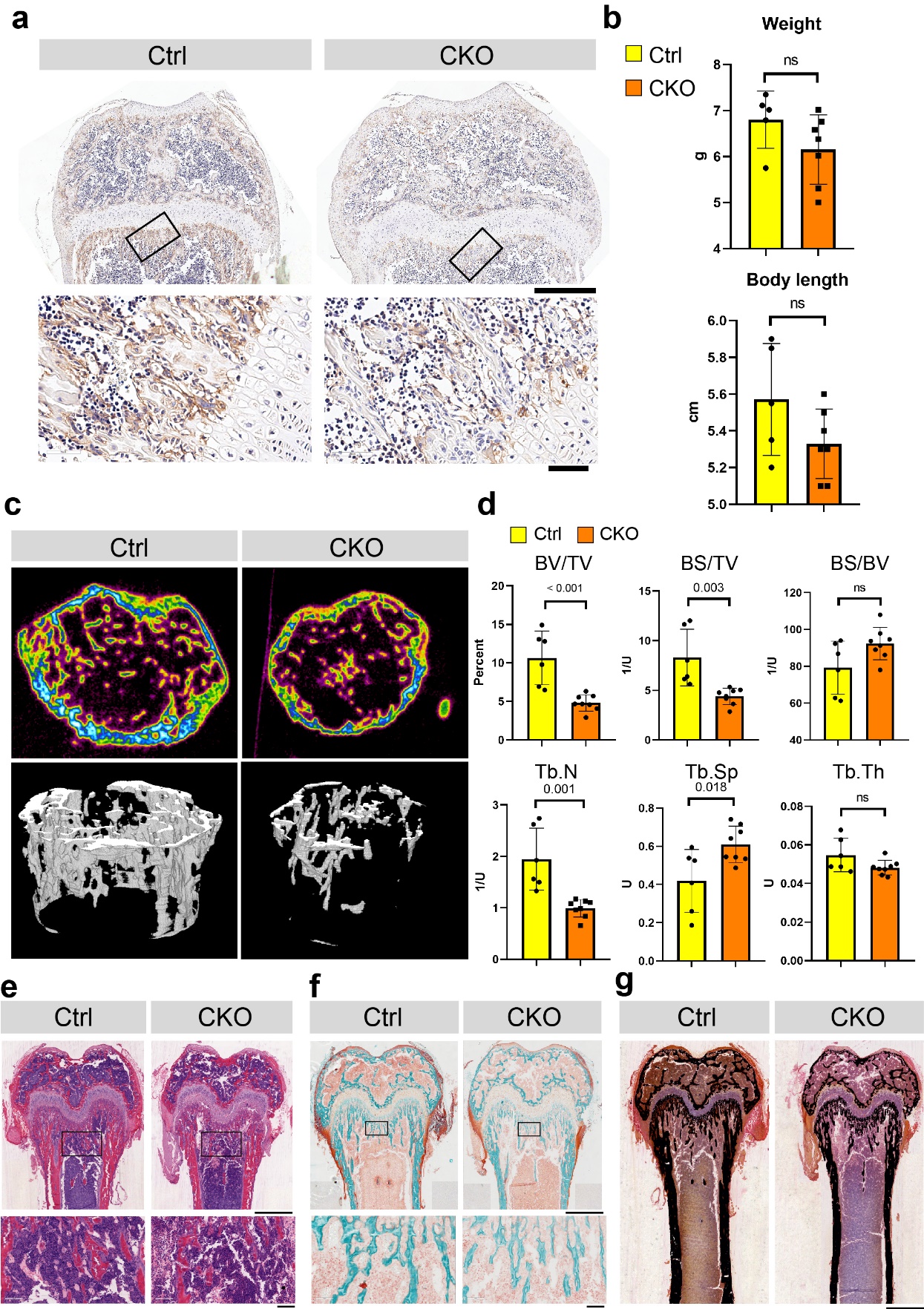
**Figure S2.** (a) Immunohistochemistry of Niban2 expressions in *Niban2* CKO male mice and its control in 4-week (scale bar: 500 μm; for magnification, scale bar: 50 μm). Representative images for 3 independent samples. (b) Weight and body length of *Niban2* CKO mice and its control in 4-week. (c) Representative cross section and reconstruction images of *Niban2* CKO mice and control mice in 4 weeks by μCT. (d) Quantitative analysis of the μCT results of panel c. (e) Representative H&E staining of Niban2 CKO mice and control mice (scale bar: 800 μm; for magnification, scale bar: 100 μm). (f) Representative Goldner trichrome staining of Niban2 CKO mice and control mice (scale bar: 800 μm; for magnification, scale bar: 50 μm). (g) Representative von Kossa staining of Niban2 CKO mice and control mice (scale bar: 800 μm). Data are presented as the mean (SD), and individual data are indicated as points. *P* values were tested by unpaired Student’s t test. *P* values were presented if it < 0.05.


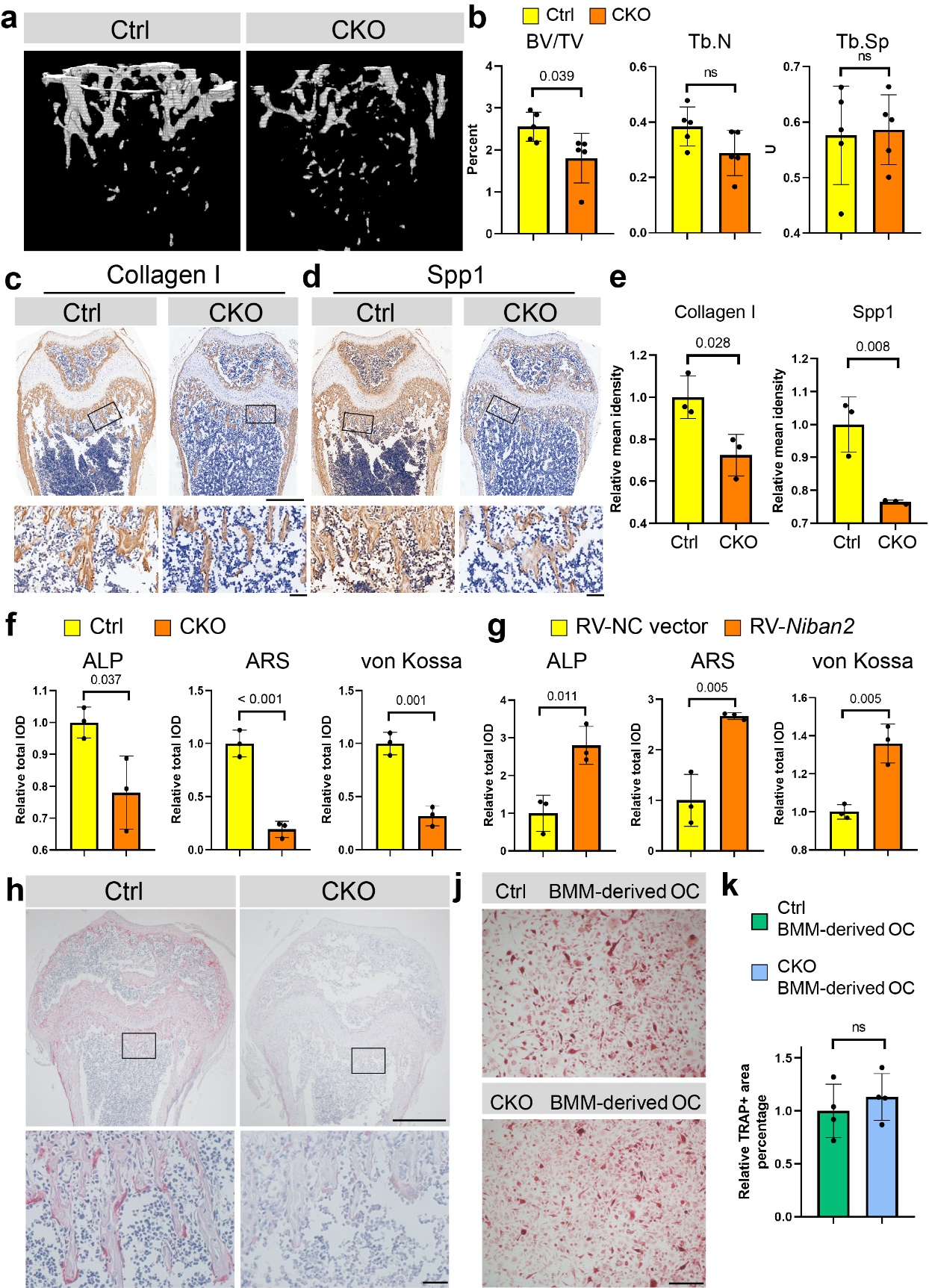
**Figure S3.** (a) Representative cross section and reconstruction images of *Niban2* CKO female mice and control mice in 8 weeks by μCT. (b) Quantitative analysis of the μCT results of panel a. Representative Collagen I (c) and Spp1 (d) immunohistochemistry staining of Niban2 CKO male mice and control mice (scale bar: 800 μm; for magnification, scale bar: 50 μm). (e) Quantitative analysis of results in panels a and b. (f-g) Quantitative analysis of the osteoblast differentiation staining of Figure 3b and Figure 3d. (h) Representative TRAP staining of *Niban2* CKO male mice and control mice (scale bar: 800 μm; for magnification, scale bar: 50 μm). (j) Representative TRAP staining of BMM-derived osteoclasts from *Niban2* CKO mice and control mice *in vitro* (scale bar: 50 μm). (k) Quantitative analysis of the TRAP staining in panel g. Data are presented as the mean (SD), and individual data are indicated as points. *P* values were tested by unpaired Student’s t test. *P* values were presented if it < 0.05.


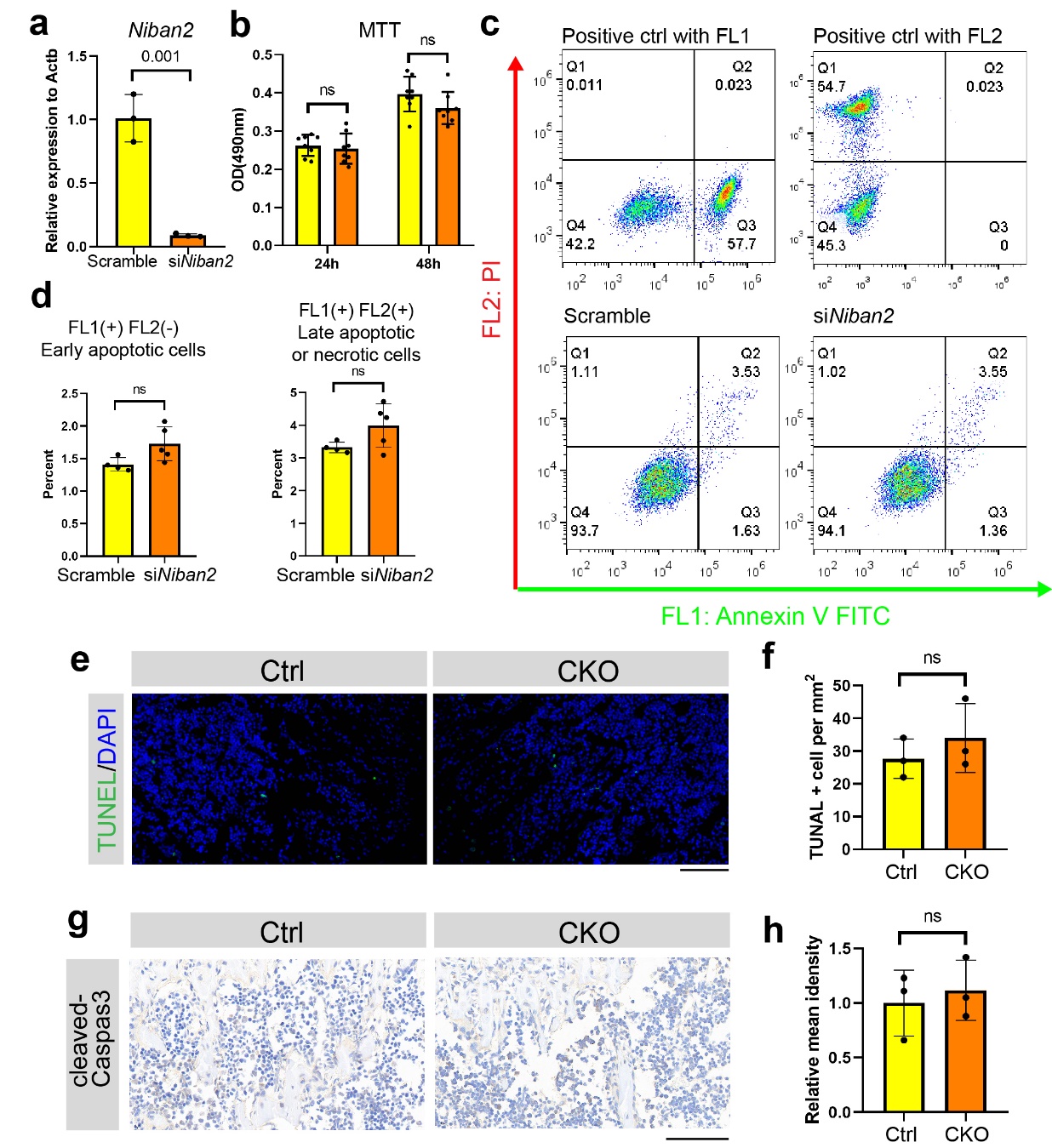
**Figure S4.** (a) Result of quantitative RT‒PCR to detect relative expression of *Niban2* in *Niban2* knockdown and the control MC3T3-E1. (b) Cell growth and viability detection with MTT in *Niban2* knockdown and the control MC3T3-E1. (c) Representative detection of apoptosis by flow cytometry with Annexin V and PI staining in *Niban2* knockdown and the control MC3T3-E1. (d) Quantitative analysis of the apoptosis in panel c. (e) TUNEL assay of Niban2 CKO male mice and control mice (scale bar: 100 μm). (f) Quantitative analysis of results in panel e. (g) Representative cleaved-caspase3 immunohistochemistry staining of Niban2 CKO male mice and control mice (scale bar: 100 μm). (h) Quantitative analysis of results in panel g. Data are presented as the mean (SD), and individual data are indicated as points. *P* values were tested by unpaired Student’s t test or one-way ANOVA followed by Bonferroni’s post hoc test. *P* values were presented if it < 0.05.


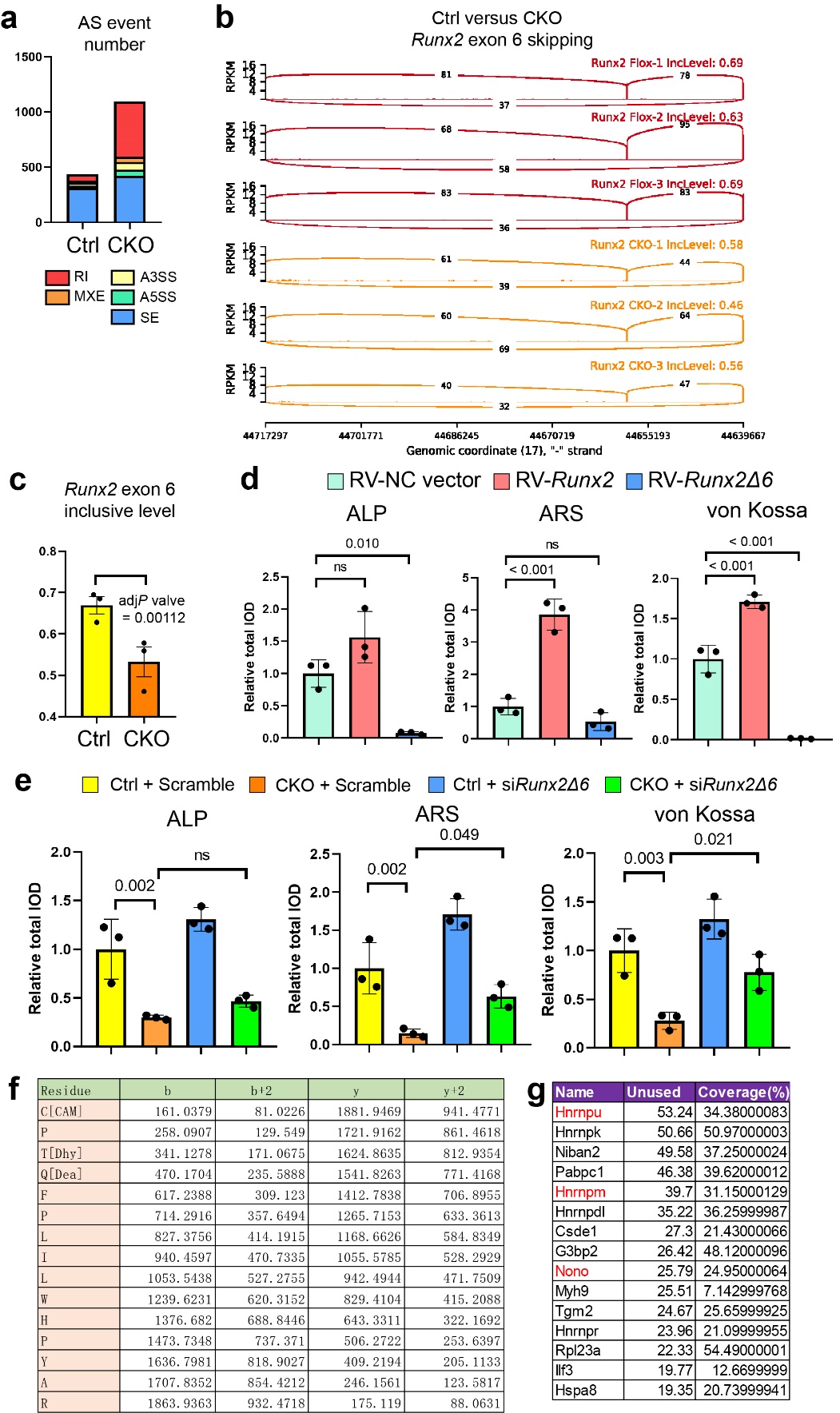


**Figure S5.** (a) Summary data of significant different alternative splicing events (calculated by rMATS) in RNA-seq dataset (Figure 3e). (b) Alternative splicing analysis of *Runx2* exon 6 skipping by rMATS in *Niban2* CKO pre-OBs and the controls. (c) Summary data and rMATS calculated p valve of Runx2 exon 6 skipping AS. (d) Quantitative analysis of the osteoblast differentiation staining of Figure 4g. (e) Quantitative analysis of the osteoblast differentiation staining of Figure 4i. (f) Peak information of Figure 5a. (g) Lists of identified proteins in Co-IP with top score of Unused in Figure 5b. Data are presented as the mean (SD), and individual data are indicated as points. *P* values were tested by one-way ANOVA followed by Bonferroni’s post hoc test. *P* values were presented if it < 0.05.


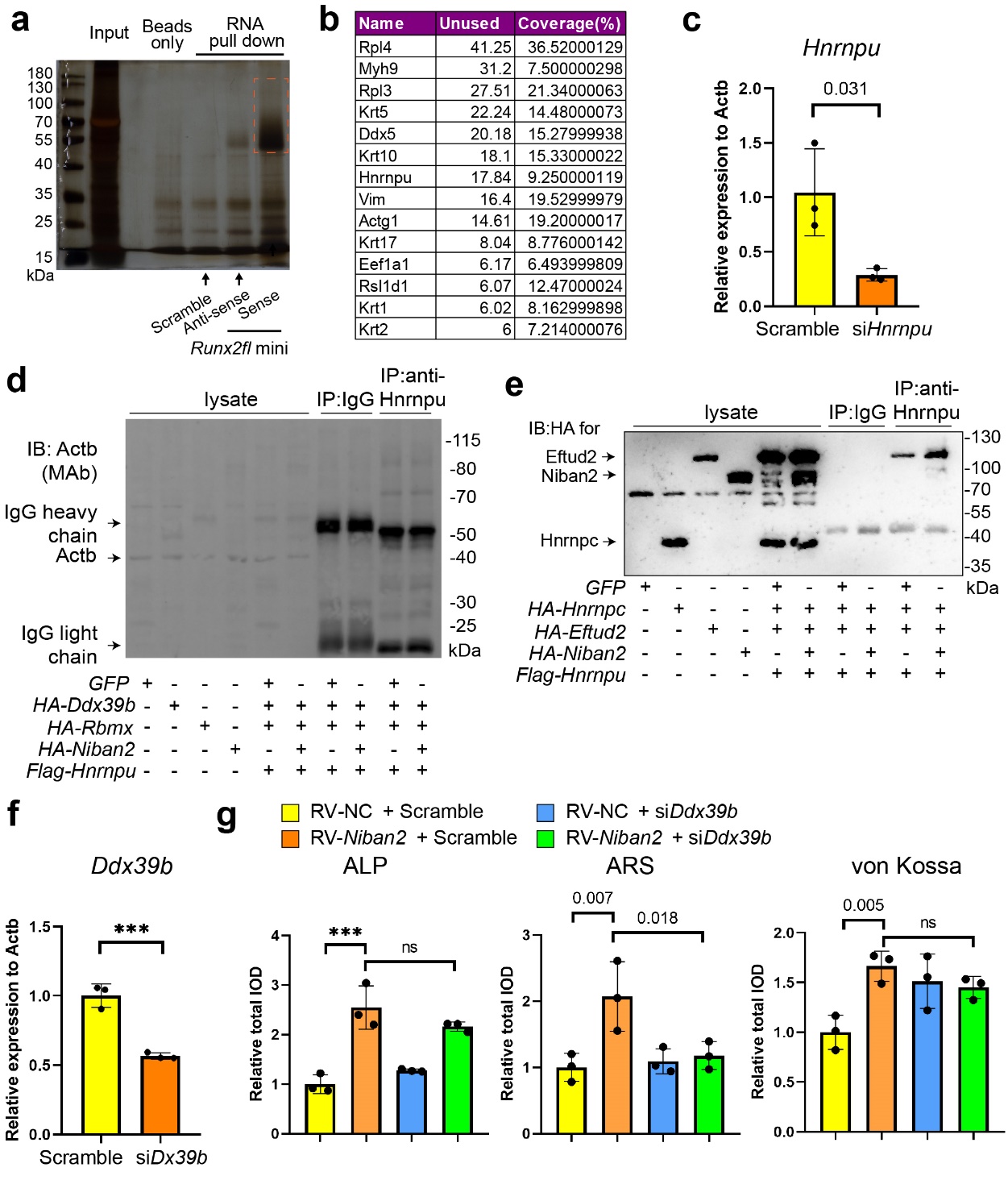


**Figure S6.** (a) Silver stain of RNA pulldown for *Runx2* exon 6 binding proteins in Figure 5f. (b) Lists of identified proteins in RNA pulldown with the top score of Unused in Figure 5f. (c) Result of quantitative RT‒PCR to detect relative expression of *Hnrnpu* in *Hnrnpu* knockdown pre-OBs and its control cells. (d) Western blots of mouse anti-Gapdh with the same blot of Figure 6f. HEK293T cells were first transfected with plasmids expressing HA-labeled spliceosomal complex components. IB was carried out to detect Niban2 and the other spliceosomal complex components, including Hnrnpc and Eftud2 (e). (f) Result of quantitative RT‒PCR to detect relative expression of *Ddx39b* in *Ddx39b* knockdown cells and the control cells. (g) Quantitative analysis of the osteoblast differentiation staining of Figure 6h. Data are presented as the mean (SD), and individual data are indicated as points. *P* values were tested by one-way ANOVA followed by Bonferroni’s post hoc test. *P* values were presented if it < 0.05.


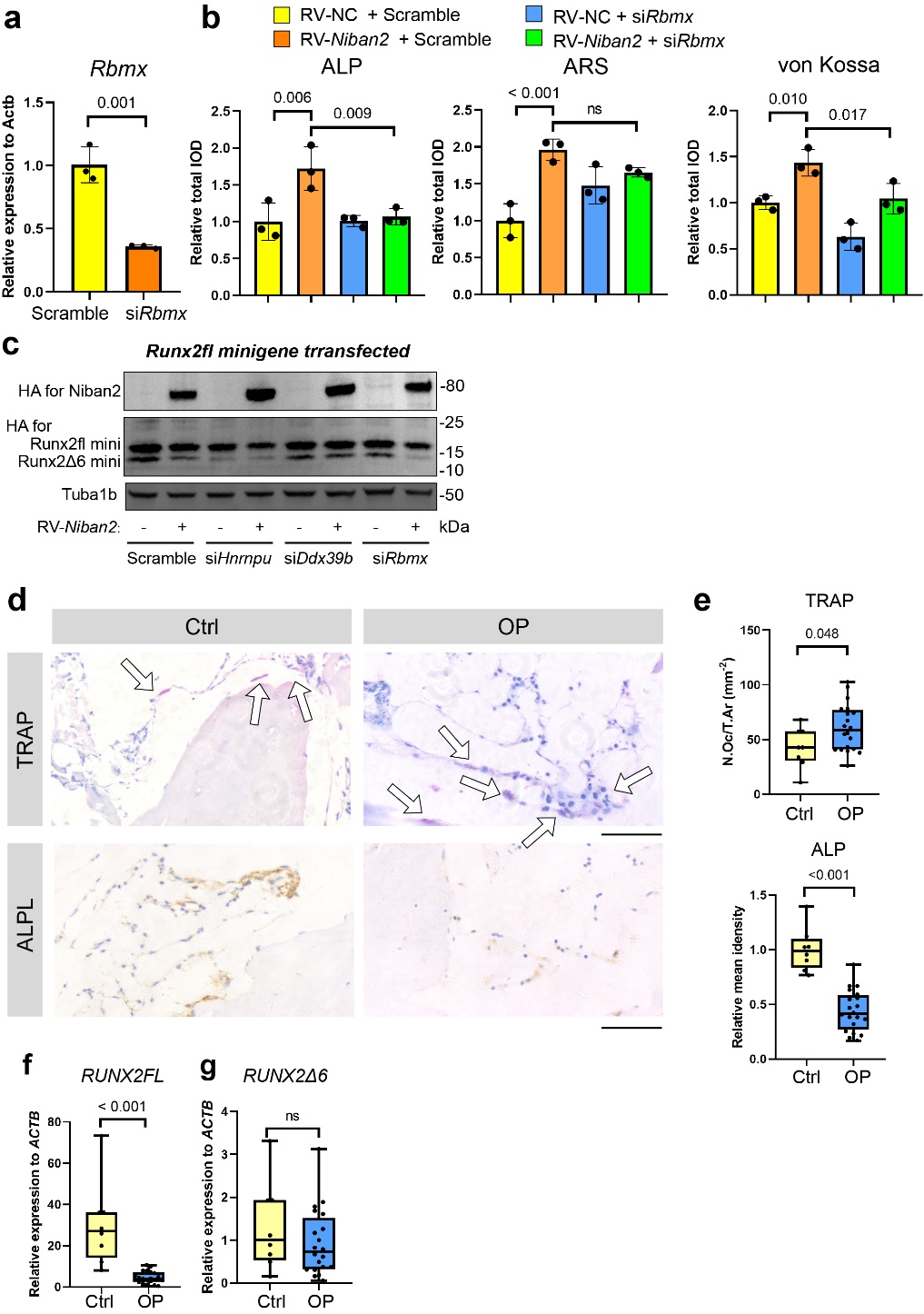


**Figure S7.** (a) Result of quantitative RT‒PCR to detect relative expression of *Rbmx* in *Rbmx* knockdown cells and the control cells. (b) Quantitative analysis of the osteoblast differentiation staining of Figure 6k. (c) Western blots of HA for Niban2 and Runx2 mini in *Niban2*-OE and its controls with transfection of *siHnrnpu*, *siDdx39b*, *siRbmx*, and scramble in *Runx2* mini expressing MC3T3-E1. (d) Representative TRAP staining and ALPL immunohistochemistry staining of osteoporosis patients and controls (scale bar: 100 μm). (e) Quantitative analysis of results in panel d. (e) Quantitative analysis of results in panel g. Relative expression of *RUNX2FL* (f) and *RUNX2Δ6* (g) to *ACTB* in osteoporosis patients and controls, respectively. *P* values were tested by unpaired Student’s t test or one-way ANOVA followed by Bonferroni’s post hoc test. *P* values were presented if it < 0.05.


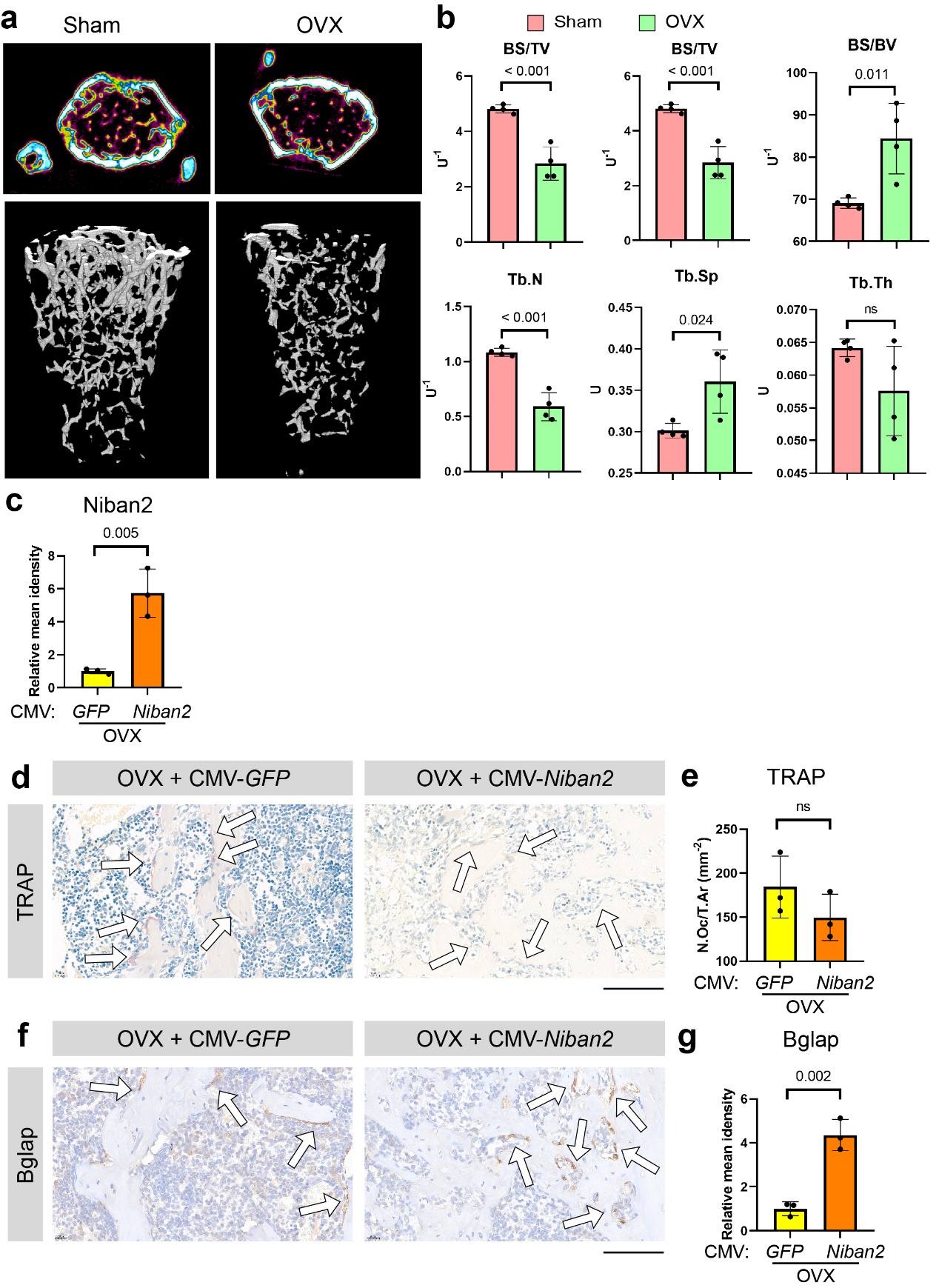


**Figure S8.** (a) Representative cross section and reconstruction images of ovariectomy mice and control mice by μCT. (b) Quantitative analysis of the μCT results of panel f. (c) Quantitative analysis of results in Figure 7h. (d) Representative TRAP staining of *Niban2*-OE feumr and its control (scale bar: 100 μm), and arrows indicated positive staining cells. (e) Quantitative analysis of results in panel d. (f) Representative Bglap immunohistochemistry staining of *Niban2*-OE feumr and its control (scale bar: 100 μm), and arrows indicated positive staining cells. (g) Quantitative analysis of results in panel f. Data are presented as the mean (SD), except min to max bar and quarters in boxplots, and individual data are indicated as points. *P* values were tested by unpaired Student’s t test. *P* values were presented if it < 0.05.

|  |  |  |  |  |
| --- | --- | --- | --- | --- |
|  |  |  | Forward (5'-3') | Reverse (5'-3') |
| Primers for qPCR | | |  |  |
|  | *Mus musculus* | |  |  |
|  |  | *Actb* | GCGGGCGACGATGCT | CACGGTTGGCCTTAGGGTT |
|  |  | *Niban2* | CCCTCTCATCCTGTGGCATC | CGAACGCAATCCTGAAGCAC |
|  |  | *Zfhx* | CAAACCCAGTAGCATGGAGCA | ATGTAGGTCTGGAGGCTGGA |
|  |  | *Fam234a* | AGATCCTGTGCTGATGAAGGC | TACAAGGGCGACATCTTGGG |
|  |  | *Hnrnpu* | TAGAGGCCGTGGAGGATTCA | GGCCACGTGGGTATGGATAG |
|  |  | *Ddx39b* | GAGGTCCAGCATGAGTGCAT | TGTGACACATCACCAGCACA |
|  |  | *Rbmx* | CACGTAGTGCTCCACCTACA | ATCAGATCGGCTTCCTCCAC |
|  |  | *Runx2*-P1 | CATGGCCGGGAATGATGAGA | GGCCACTTGGGGAGGATTTG |
|  |  | *Runx2*-P2 | GAACCAAGAAATCCCAGGCAGG | GTTGGAGAAGCGGCTCTCAG |
|  |  | *Runx2*-P3 *(exon 6)* | TCTGACCGCCTCAGTGATTT | AAGGACTTGGTGCAGAGTTCA |
|  | *Homo sapiens* | |  |  |
|  |  | *ACTB* | CATGTACGTTGCTATCCAGGC | CTCCTTAATGTCACGCACGAT |
|  |  | *NIBAN2* | CTGACGGAGTTCCTCCAGTTC | GAGGTTCCCCGAGAAGACGAT |
|  |  | *RUNX2Δ6* | GAACCAAGAAATCCCAGGCAGG | GTTGGAGAAGCGGCTCTCAG |
|  |  | *RUNX2FL (exon 6)* | TCTGACCGCCTCAGTGATTT | AAGGACTTGGTGCAGAGTTCA |
| Primers for RIP-qPCR | | |  |  |
|  |  | *ACTB exon 4* | AGGGAAATCGTGCGTGACAT | GGAAAAGAGCCTCAGGGCAT |
|  |  | *RUNX2 exon 6* | TCTGACCGCCTCAGTGATTT | AAGGACTTGGTGCAGAGTTCA |
| siRNA |  |  |  |  |
|  |  | *siNiban2* | CUUACGCCCGACACUAUUATT | UAAUAGUGUCGGGCGUAAGTT |
|  |  | *siRunx2Δ6* | CGGGAACCAAGAAAUCCCATT | UGGGAUUUCUUGGUUCCCGTT |
|  |  | *siHnrnpu* | GUUGCUUGGUGAAGAAGAATT | UUCUUCUUCACCAAGCAACTT |
|  |  | *siDdx39b* | CUGGUGAUGUGUCACACUATT | UAGUGUGACACAUCACCAGTT |
|  |  | *siRbmx* | GUCUAUCAAGAAGGACGAATT | UUCGUCCUUCUUGAUAGACTT |

**Table S1** Oligos used in this research.

Supplementary Methods

**Isolation and culture of pre-osteoblasts.** In brief, mechanically dissociated calvaria were digested with collagenase II (1 mg/ml; Gibco, USA, 17101015) and dispase II (2 mg/ml; Millipore Sigma, USA, D4693) in serum-free α-MEM (HyClone, USA, SH30265.01B) supplemented with DNase I (100 U/mL; BioFroxx, Germany, 1121MG010) and incubated at 37°C for 1 hour under constant agitation. Cells were cultured in α-MEM with 10% fetal bovine serum (Gibco, USA, 10099141), 1% penicillin‒streptomycin (Procell, China, PB180122), and 2 mM L-glutamine (Procell, China, PB180420) at 37°C with 5% CO_2_.

**Cell lines.** The mouse pre-OB cell line MC3T3-E1 subclone 14 was purchased from ATCC (CRL-2594) and maintained in DMEN/F12 (Hyclone, USA, SH30023.01) with 10% fetal bovine serum (Gibco, USA, 10099141) and 1% penicillin‒streptomycin (Procell, China, PB180122). Cells were cultured at 37°C with 5% CO_2_. HEK293T cells (ATCC CRL-3216) were used in this research for lentivirus and retrovirus packaging and some experiments requiring transfection or large numbers of cells.

**Osteoblast differentiation.** All the OB differentiation experiments were performed with cells in 24-wells or 48-wells cell plates. After seeding into gelatin-embedded plates, primary pre-OBs or pre-OB cell line were switched to OB differentiation medium. The OB differentiation medium applied in this research was generated with 10 nM dexamethasone (Sigma‒Aldrich, USA, D2915), 10 mM β-glycerophosphate (Sigma‒Aldrich, USA, G6251), and 50 µM L-ascorbic acid (Sigma‒Aldrich, USA, A4403) to the above mentioned complete culture medium. For OB differentiation staining groups, staining was performed with 1% alizarin red S solution (Sigma‒Aldrich, USA, A5533), an ALP staining kit (Beyotime, China, C3206), and a von Kossa staining kit (Servicebio, China, GP1054) after induction for the relative days described in the legends. Whole-well images of staining were captured with a camera (Nikon, Japan, D100). For the quantitative analysis, the area and integral optical density for corresponding positive staining were calculated by Image-Pro Plus (Media Cybernetics, USA).

**Biomechanical analysis.** For the mechanical analysis of the samples, the proximal and distal ends of the tibias were embedded in epoxy to load the universal testing machine (Quanlitest, China, WAW-600C). The press test was conducted to examine the maximum load and deformation with the load point in displacement control moving at a rate of 0.05 mm/s with force and displacement data collected at 60 Hz.

**Immunohistochemistry.** Immunohistochemical staining of paraffin sections was performed according to a previously described protocol with modifications [1]. Antigen retrieval was performed with citrate buffer solution (pH 6.0) at 95°C for 5 min with three repeats. The nuclei were counterstained using hematoxylin. Images were captured and analyzed by a semiautomatic histology system (Leica, China, Aperio VERSA 8). The additional information for antibodies and other materials in this paper can be found in *SI appendix*, *Supplementary Methods*.

**multiplex** **immunofluorescence (mIF).** mIF was performed with the commercial kit (Absin, China, abs50029) on frozen sections following the manual. Briefly, sections were firstly carried out with rehydration, H_2_O_2_ treatment, permeability and block. Then after first primary antibodies and poly HRP-labeled secondary antibodies were incubated, tyramide signal amplification (TSA) reaction was activated to label the first panel. After previous antibodies complex was removed by stripping buffer for mIF (abs994), next round of incubation and reaction was performed until all the panels were labelled (TSA-520, TSA-570, TSA-620, and TSA-700). Sections were autofluorescence quenched and mounted with DAPI (Vector, USA, SP-8400). mIF images were acquired by Vectra3 imaging system (Akoya Biosciences, USA) and unmixed by Inform 2.6 (Akoya Biosciences, USA). The cell segmentations were performed with a custom training model created by Cellpose2 [2] with mIF data in this research and quantified by ImageJ (<https://imagej.net/>).

**Immunohistochemistry.** Immunohistochemical staining of paraffin sections was performed according to a previously described protocol with modifications [1]. Antigen retrieval was performed with citrate buffer solution (pH 6.0) at 95°C for 5 min with three repeats. After incubation with primary antibodies, biotinylated secondary antibodies (Zhongshan Biotechnologies, China, PV6001) were incubated, and a 3,3-diaminobenzidine tetrahydrochloride-based system (Zhongshan Biotechnologies, China, ZLI-9017) was applied for detection. The nuclei were counterstained using hematoxylin. Immunochemistry was performed using antibodies against Niban2 (Novus Biologicals, USA, NBP1-88784), Col1a1 (Affinity Biosciences, China, AF7001), Spp1 (Proteintech group, China, 22952-1-AP), Alpl (Servicebio, GB11527), Bglap (Servicebio, GB120012), cleaved-caspase3 (Servicebio, GB115733). Images were captured and analyzed by a semiautomatic histology system (Leica, China, Aperio VERSA 8).

**Immunofluorescence.** Immunofluorescence staining of cells was performed following a previous protocol with modifications [3]. For cell samples, fixation in 4% paraformaldehyde for 30 min at room temperature was first processed. After incubation with primary antibodies and fluorescence-labeled secondary antibodies (Abcam, UK, ab150077), the cells were stained with DAPI (Servicebio, China, G1012). Immunofluorescence was performed using antibodies against Niban2 (Novus Biologicals, USA, NBP1-88784). Images of sections and cell samples were captured with confocal microscopy (Leica, Germany, SP8).

**Fluorochrome labeling.** For labeling bone mineralization, 25 mg/kg calcein (Sigma‒Aldrich, USA, C0875) and 50 mg/kg alizarin-3-methyliminodiacetic acid (Sigma‒Aldrich, USA, A3882) dissolved in 2% sodium bicarbonate solution were subcutaneously injected into mice at 28-day intervals. Then, undecalcified femur samples were processed for solid tissue sectioning following the protocol mentioned above. Fluorochrome-labeled sections were captured by a semiautomatic histology system (Leica, Germany, Aperio VERSA 8).

**RNA fluorescence in situ hybridization.** RNA-FISH was performed with the commercial kit (Servicebio, China, GF003) on decalcified paraffin sections following the manual. The IF550-labelled probe was synthesized by Servicebio (China) to detect *Runx2* exon 6.

**Minigene reporter plasmid construction.** The construction of the *Runx2Δ6* splicing reporter minigene included *Runx2* exon 6, 150 bp of flanking intronic sequence, and the conjugated part of *Runx2* *exon* 5 and *exon* 7 (100 bp for each exon) according to a previous protocol [4]. The above sequence was synthesized by Tsingke Biotechnology (China) and cloned into pHAGE. The correct construction of the plasmid was verified by sequencing for proper in-frame translation. The plasmid transfection was performed as mentioned above.

**RNA pulldown.** PCR with primers containing the T7 promotor for linearized DNA with *Runx2fl mini* and part of its nearby intron was performed with the *Runx2Δ6* splicing reporter minigene plasmid. Then, biotin-labeled linearized RNA was synthesized with a RiboTM RNAmax-T7 transcription kit (Ribobio, China, C11002). RNA pulldown was conducted by a Pierce Magnetic RNA‒Protein Pull-Down Kit (Thermo Fisher, USA, 20164) following the manufacturer’s instructions, with scramble biotin-labeled RNA in the kit serving as a control. Pulldown proteins were then identified by MS and verified by Western blots as described above.

**Mass spectrometry for protein identification.** After sample preparation, including enzymolysis in protein gel and zip-tip desalting, peptide samples were diluted on the machine buffet. Then, the samples were loaded onto the mass spectrometer with data collection using the Triple TOF 5600 + LC/MS system (AB Sciex, USA) coupled with the Eksigent nanoLC system (AB Sciex, USA). A C18 capture column (AB Sciex, USA, 3 μm, 350 μm×0.5 mm) and C18 analytical column (AB Sciex, USA, 3 μm, 75 μm×150) were applied in the process. For protein identification, the Paragon algorithm in ProteinPilot was used to search the UniProt database [5]. The parameters were set as follows: the instrument was a TripleTOF 5600, cysteine was modified with iodoacetamide, and biological modification was selected as the ID focus. For the identified protein results, certain filtering criteria were selected, peptides with an unused score> 1.3 (a credibility of more than 95%) were considered credible peptides, and proteins containing at least one unique protein were excluded. Mass spectrometry analysis for protein identification was performed by Wuhan GeneCreate Biological Engineering Co., Ltd. (China).

**scRNA-seq analysis.** Osteolineage scRNA-seq data were downloaded from GSM2915579, and Col2.3^+^ cells were selected for further analysis. Seurat was applied to perform quality control, normalization, clustering, and data visualization [6]. Pseudotime trajectory analysis was processed by Monocle3 following the instructions [7]. GSVA analysis for scRNA-seq was performed first by calculating pathway variation at the single-cell level and then displaying the average variation level in each cluster [8].

**RNA-seq and analysis.** After the generation of *Niban2* CKO pre-OBs and their controls, total RNA was first prepared using a total RNA extraction kit (Aidlab Biotech, China, RN07), and then, mRNA was purified by an oligo dT library kit for RNA-seq analysis following the manufacturer’s instructions (Illumina, USA, HiSeq 4000). Sequencing reads were mapped to the *Mus musculus* genome GRCm38 using STAR [9], and tag counts were summarized at the gene level using SAMtools [10], which allowed only one read per position per length. Differentially expressed genes (DEGs) were determined by DESeq2 [11]. DEGs were used for heatmap analysis using the R package pheatmap (https://github.com/raivokolde/pheatmap). Gene set enrichment analysis, including GO and KEGG analyses, was performed using DEGs with clusterProfiler [12]. GO analysis was visualized by GOplot [13]. RNA-seq data from GSE113253 and GSE202080 were also reanalyzed following the above process. Alternative splicing analysis was performed with the package rMATS following the general process [14]. The inclusion level was calculated by the following formula:

$$\varphi={(I/l_{I})}/{(I/l_{I}+S/l_{S})}$$

Symbol definitions and explanations can be found in the online tutorial. (<https://github.com/Xinglab/rmats-turbo/blob/v4.1.2/README.md>)

**RNA Binding Protein Immunoprecipitation (RIP).** RIP was processed as previously described [15]. Briefly, HEK293T cells were transfected with plasmids following the group information in the figure legend. Then, the cells were crosslinked with formaldehyde solution, and the cell lysates were processed for immunoprecipitation using antibodies conjugated to magnetic beads against FLAG. The captured RNA was detected by quantitative RT‒PCR following the process mentioned above.

**Antibodies information.** mIF was performed using antibodies against Niban2 (Novus Biologicals, USA, NBP1-88784), Sp7 (Abcam, UK, ab209484), Runx2 (Cell Signaling Technology, USA, 12556), and Bglap (Santa Cruz, USA, sc-365797). Antibodies for western blots were following listed. Antibodies against Niban2 (NBP1-88784) were purchased from Novus Biologicals (USA). Antibodies against Runx2 (12556) were purchased from Cell Signaling Technology (USA). Antibodies against Sp7 (ab209484) were purchased from Abcam (UK). Antibodies against Tuba1 (11224-1-AP) and HA from rabbit hosts (51064-2-AP) were purchased from Proteintech (China). Antibodies against HA from a mouse host (T0008) were purchased from Affinity Biotechnology (China).

**Reference**

[1] Y. Han, H. Feng, J. Sun, X. Liang, Z. Wang, W. Xing, Q. Dai, Y. Yang, A. Han, Z. Wei, Q. Bi, H. Ji, T. Kang, W. Zou, *The Journal of clinical investigation* **2019**, *129* (5), 1895, <https://doi.org/10.1172/jci124590>.

[2] M. Pachitariu, C. Stringer, *Nat Methods* **2022**, *19* (12), 1634, <https://doi.org/10.1038/s41592-022-01663-4>.

[3] A. P. Kusumbe, S. K. Ramasamy, A. Starsichova, R. H. Adams, *Nature protocols* **2015**, *10* (12), 1904, <https://doi.org/10.1038/nprot.2015.125>.

[4] P. Gaildrat, A. Killian, A. Martins, I. Tournier, T. Frébourg, M. Tosi, *Methods in molecular biology (Clifton, N.J.)* **2010**, *653*, 249, <https://doi.org/10.1007/978-1-60761-759-4_15>.

[5] *Nucleic acids research* **2021**, *49* (D1), D480, <https://doi.org/10.1093/nar/gkaa1100>.

[6] A. Butler, P. Hoffman, P. Smibert, E. Papalexi, R. Satija, *Nature biotechnology* **2018**, *36* (5), 411, <https://doi.org/10.1038/nbt.4096>.

[7] C. Trapnell, D. Cacchiarelli, J. Grimsby, P. Pokharel, S. Li, M. Morse, N. J. Lennon, K. J. Livak, T. S. Mikkelsen, J. L. Rinn, *Nature biotechnology* **2014**, *32* (4), 381, <https://doi.org/10.1038/nbt.2859>.

[8] S. Hänzelmann, R. Castelo, J. Guinney, *BMC bioinformatics* **2013**, *14*, 7, <https://doi.org/10.1186/1471-2105-14-7>.

[9] A. Dobin, C. A. Davis, F. Schlesinger, J. Drenkow, C. Zaleski, S. Jha, P. Batut, M. Chaisson, T. R. Gingeras, *Bioinformatics (Oxford, England)* **2013**, *29* (1), 15, <https://doi.org/10.1093/bioinformatics/bts635>.

[10] H. Li, B. Handsaker, A. Wysoker, T. Fennell, J. Ruan, N. Homer, G. Marth, G. Abecasis, R. Durbin, *Bioinformatics (Oxford, England)* **2009**, *25* (16), 2078, <https://doi.org/10.1093/bioinformatics/btp352>.

[11] M. I. Love, W. Huber, S. Anders, *Genome biology* **2014**, *15* (12), 550, <https://doi.org/10.1186/s13059-014-0550-8>.

[12] G. Yu, L. G. Wang, Y. Han, Q. Y. He, *Omics : a journal of integrative biology* **2012**, *16* (5), 284, <https://doi.org/10.1089/omi.2011.0118>.

[13] W. Walter, F. Sánchez-Cabo, M. Ricote, *Bioinformatics (Oxford, England)* **2015**, *31* (17), 2912, <https://doi.org/10.1093/bioinformatics/btv300>.

[14] S. Shen, J. W. Park, Z. X. Lu, L. Lin, M. D. Henry, Y. N. Wu, Q. Zhou, Y. Xing, *Proceedings of the National Academy of Sciences of the United States of America* **2014**, *111* (51), E5593, <https://doi.org/10.1073/pnas.1419161111>.

[15] M. Gagliardi, M. R. Matarazzo, *Methods in molecular biology (Clifton, N.J.)* **2016**, *1480*, 73, <https://doi.org/10.1007/978-1-4939-6380-5_7>.
